# Aggregation-induced emission from optically active X-shaped molecules based on planar chiral [2.2]paracyclophane

**DOI:** 10.1038/s41598-023-49120-2

**Published:** 2023-12-19

**Authors:** Keishi Jikuhara, Ryo Inoue, Yasuhiro Morisaki

**Affiliations:** Department of Applied Chemistry for Environment, School of Biological and Environmental Sciences, Kwansei Gakuin University, 1 Gakuen Uegahara, Sanda, Hyogo 669-1330 Japan

**Keywords:** Optical materials, Stereochemistry

## Abstract

An optically active π-stacked molecule was synthesized incorporating planar chiral [2.2]paracyclophane and *o*-carborane units to impart circularly polarized luminescence and aggregation-induced emission properties to the molecule. The molecule exhibited a strong emission from the aggregated state in a mixed solvent system (H_2_O/THF) and the solid state in the PMMA matrix. In the aggregated state, weak circularly polarized luminescence was observed owing to the random intermolecular orientation. On the other hand, the circularly polarized luminescence was clearly observed in the PMMA film containing 1 wt% molecule. Theoretical studies using time-dependent density functional theory reproduced the molecule’s circular dichroism and circularly polarized luminescence properties.

## Introduction

Aggregation-induced emission (AIE) is a well-known emission phenomenon^1–3^, in which molecules are emissive upon aggregate formation and are not emissive in their solution states. Generally, fluorophores exhibit strong emission in the isolated states, such as in solution, and aggregation-caused quenching (ACQ) occurs in the aggregates, solids, and crystals. In 2001, Tang et al. reported that 1-methyl-1,2,3,4,5-pentaphenylsilole was effectively emissive in the aggregated state despite the absence of fluorescence in the diluted solution^4^. Therefore, researchers have prepared a wide variety of molecules exhibiting AIE using various AIE-active units, such as arylenevinylenes^5,6^ in addition to the pentaphenyl siloles^4^. A typical AIE mechanism arises from the suppressing molecular motions in the aggregates. Interference of intermolecular interactions among π-conjugated fluorophores allows to generate bright AIE. In 2008, Chujo reported that *o*-carborane-based π-conjugated molecules exhibited the AIE properties^7^. When the stretching vibration of the carbon–carbon bond in the *o*-carborane unit^8^ is frozen by aggregation, the non-radiative decay of the π-conjugated units is suppressed, leading to AIE from the aggregates and solids. This emission is a charge-transfer (CT) process between the electron-rich π-conjugated moieties substituted directly at the carbon of *o*-carborane and the electron-poor *o*-carborane unit. The significance of the dihedral angle between the carbon–carbon bond in the *o*-carborane unit and π-conjugated plane has also been reported^9,10^.

[2.2]Paracyclophane is a cyclic compound in which two phenylene groups are stacked in close proximity^11–13^. Since its discovery^14^ and practical synthesis^15^, various [2.2]paracyclophane derivatives have been synthesized. The rotatory motion of the phenylenes in [2.2]paracyclophane is entirely suppressed because of the closely stacked phenylenes; therefore, [2.2]paracyclophanes with substituent(s) become planar chiral compounds depending on the substitution position(s)^16–27^. Planar chiral [2.2]paracyclophane derivatives have been utilized as chiral ligands and chiral auxiliaries in the fields of organometallic and organic chemistry. However, until recently, they were not employed in the fields of polymer and materials chemistry. In 2012, the synthesis of conjugated polymers bearing planar chiral pseudo-*ortho*-disubstituted [2.2]paracyclophane repeating units was reported^28^. The obtained optically active polymer emitted circularly polarized luminescence (CPL) with a relatively high anisotropy factor (*g*_lum_ value; *g*_lum_ = 2(*I*_left_ − *I*_right_)/(*I*_left_ + *I*_right_), where *I*_left_ and *I*_right_ are photoluminescence intensities of the left-handed and right-handed CPL)^29–31^ of the order of 10^−3^. A variety of optically active conjugated molecules, such as polymers^28,32,33^, macrocycles^34,35^, and π-stacked dimers^36–39^ comprising planar chiral [2.2]paracyclophanes, have been prepared, and their CPL behaviors have been investigated in detail. The researches on the CPL-emitters using chiral scaffolds including planar chiral [2.2]paracyclophanes have started a short time, and it is meaningful to prepare solid CPL emissive materials by combining AIEgens and chiral building blocks and to elucidate their emission behaviors for promising applications in three-dimensional display device, security ink, plant growth promoting film, biological imaging material, and so on^40–42^. In this study, *o*-carborane and planar chiral [2.2]paracyclophane were fused in a molecule, creating a conjugated molecule exhibiting aggregation-induced CPL. *o*-Carborane and planar chiral [2.2]paracyclophane units afforded AIE and CPL properties of the π-conjugated moiety, respectively. This research provides insights into the synthetic routes and optical properties of the novel molecule.

## Results and discussions

**Figure 1:**
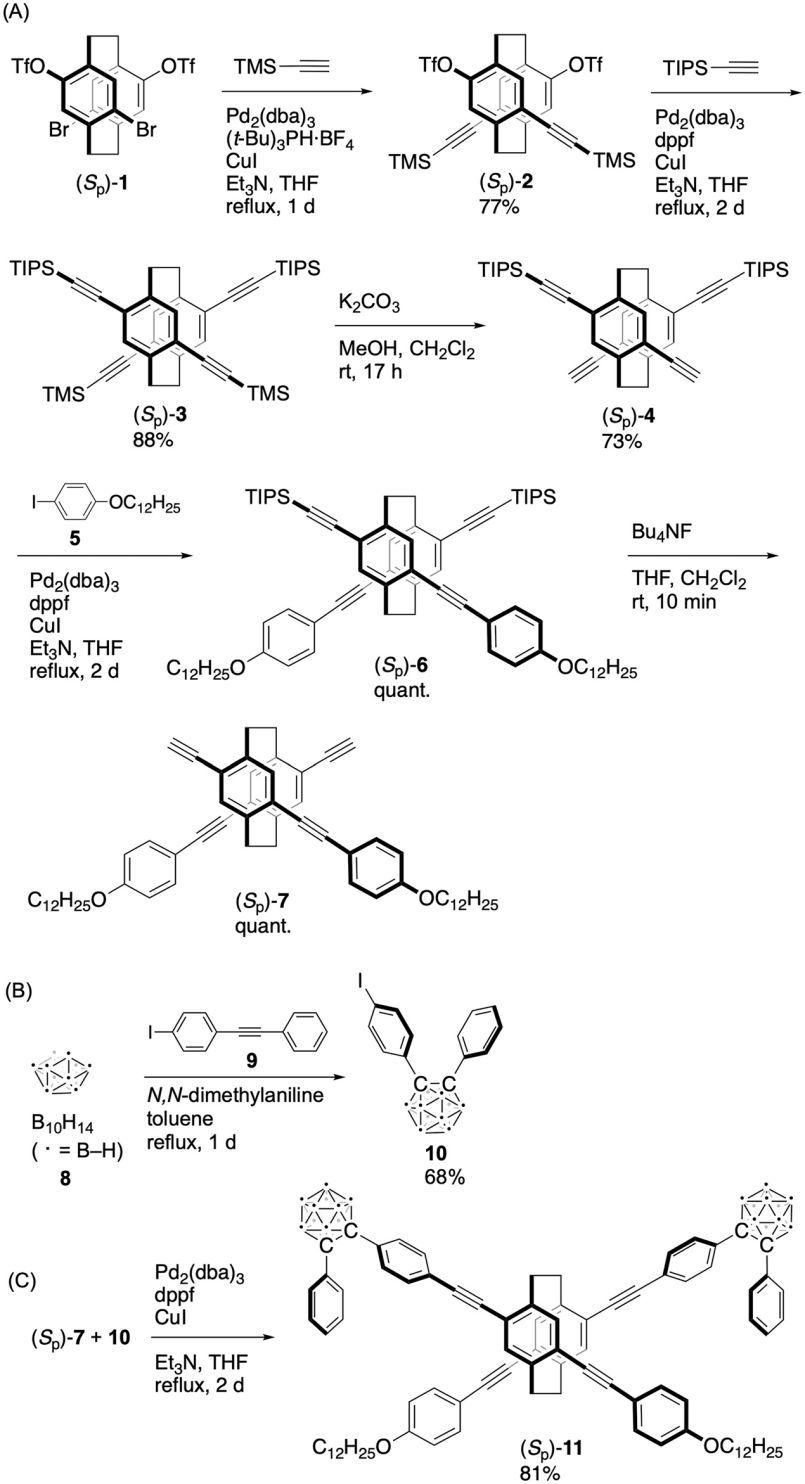
Synthesis of the optically active X-shaped molecule consisting of planar chiral [2.2]paracyclophane and *o*-carboranes.

Figure 1 illustrates the synthetic route for the (*S*_p_)-isomer of the target molecule. Optically active bis-(*para*)-pseudo-*meta*-tetrasubstituted [2.2]paracyclophane (*S*_p_)-**1** was prepared according to a previously reported method^43^. The bis-(*para*)-pseudo-*meta*-isomer was chosen instead of the corresponding pseudo-*ortho*-isomer attributed to the bulky *o*-carborane moieties located at pseudo-*meta*-positions. The chemoselective Sonogashira-Hagihara coupling^44,45^ of (*S*_p_)-**1** with trimethylsilylacetylene (TMS-acetylene) using a Pd_2_(dba)_3_/^*t*^Bu_3_P/CuI catalytic system (dba = dibenzylideneacetone) afforded (*S*_p_)-**2** in 77% isolated yield (Fig. 1A); in this reaction, bromo groups were reacted selectively. The remaining trifluoromethanesulfonyl (TfO) groups were reacted with triisopropylsilylacetylene (TIPS-acetylene) in the presence of a catalytic amount of Pd_2_(dba)_3_, 1,1'-bis(diphenylphosphino)ferrocene (dppf), and CuI to afford (*S*_p_)-**3** in 88% isolated yield. The TMS groups were selectively removed by reacting (*S*_p_)-**3** with K_2_CO_3_ and MeOH to yield (*S*_p_)-**4** (77%). Sonogashira-Hagihara coupling of (*S*_p_)-**4** with 4-dodecyloxy-1-iodobenzene **5** afforded (*S*_p_)-**6** quantitatively. The dodecyloxy (OC_12_H_25_) group was incorporated to enhance the solubility and film-forming ability of the target molecule. The TIPS groups were removed by reacting (*S*_p_)-**6** with Bu_4_NF to afford (*S*_p_)-**7** quantitatively (Fig. 1A). Finally, Sonogashira-Hagihara coupling of (*S*_p_)-**7** with iodo-substituted *o*-carborane compound **10** (Fig. 1C), which was obtained in 68% isolated yield by the reaction of decaborane (B_10_H_14_) **8** with *p*-iodotolane **9** (Fig. 1B), afforded the target molecule (*S*_p_)-**11** in 81% isolated yield. The (*R*_p_)-isomer (*R*_p_)-**11** was prepared following the same procedure as for (*S*_p_)-**11** from enantiopure (*R*_p_)-**1**. The structures of new compounds were confirmed by NMR spectroscopy and high-resonance mass (HRMS) spectrometry. Optical and chiroptical properties were investigated by specific rotation, ultraviolet–visible (UV–vis) absorption, PL, circular dichroism (CD) and CPL spectroscopy. Toxicity and/or biological activity of **11** for biological imaging applications were not investigated in the present stage.

**Figure 2:**
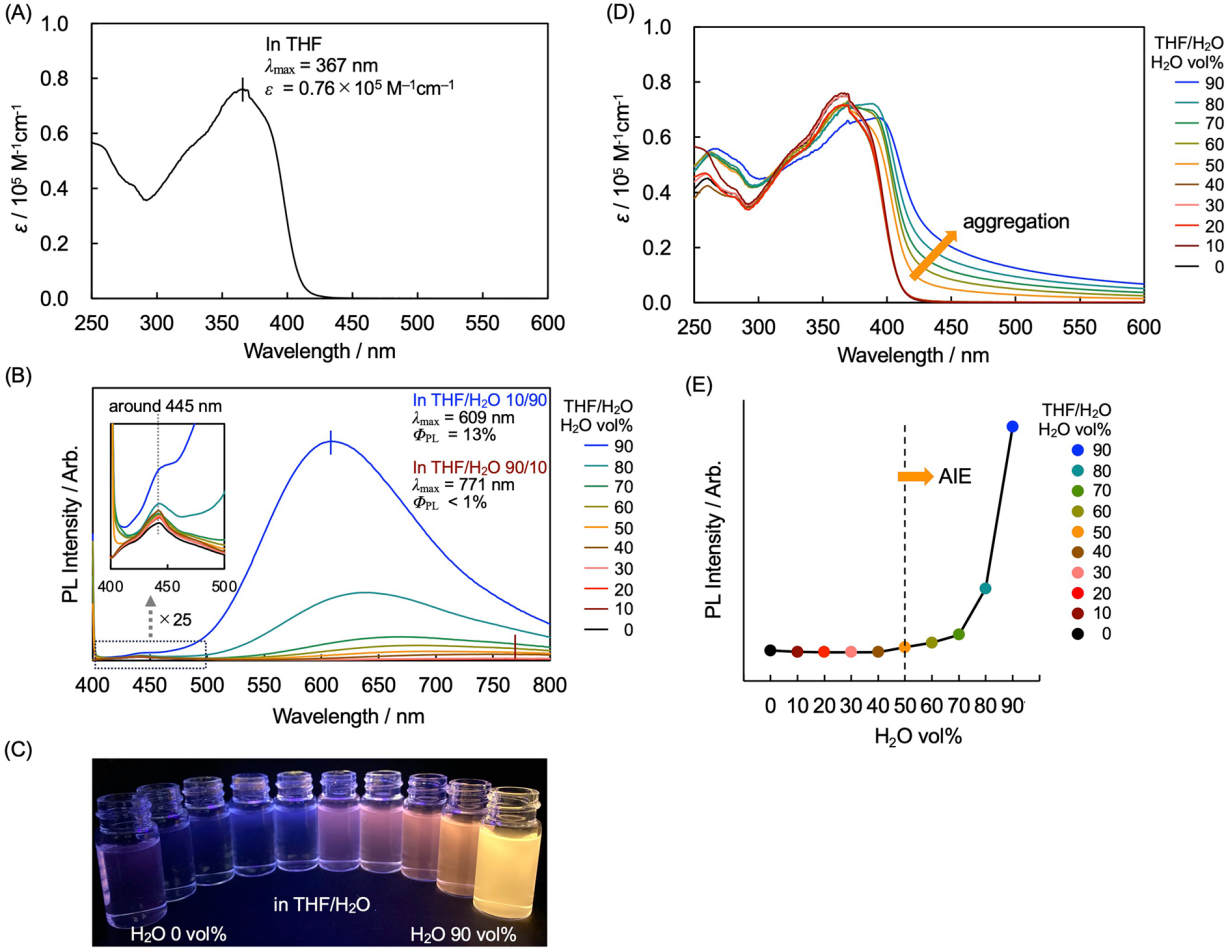
(**A**) UV–vis absorption spectrum of (*S*_p_)-**11** in THF (1.0 × 10^–5^ M). (**B**) PL spectra of (*S*_p_)-**11** in THF/H_2_O (1.0 × 10^–5^ M) excited at each absorption maximum. Inset: expanded view of the vertical axis from 400 to 500 nm. (**C**) PL in THF/H_2_O (1.0 × 10^–5^ M) excited using a handy UV lamp (*λ* = 365 nm). (**D**) UV–vis absorption spectra of (*S*_p_)-**11** in THF/H_2_O (1.0 × 10^–5^ M). (**E**) Dependence of PL intensity at *λ*_max_ of each emission peak on the solvent composition (H_2_O vol% of THF/H_2_O).

Figure 2A shows the UV–vis absorption spectrum of (*S*_p_)-**11** in tetrahydrofuran (THF) (1.0 × 10^–5^ M), revealing the π-π* absorption band of the phenylene-ethynylene moiety with molar absorption coefficient (*ε*) of 0.76 × 10^–5^ M^–1^ cm^–1^ at absorption maximum (*λ*_max_) of 367 nm. The photoluminescence (PL) spectra of (*S*_p_)-**11** in a mixed solution (1.0 × 10^–5^ M) of THF and H_2_O are shown in Fig. 2B; THF and H_2_O are good and poor solvents for (*S*_p_)-**11**, respectively. Weak emission was observed in 100% THF solution, whereas a strong emission peak was observed (peak top *λ*_max_ = 609 nm) in the THF/H_2_O mixed solution (H_2_O 90 volume% = vol%) with a PL quantum yield (*Φ*_PL_) of 13%. Figure 2C shows their PL excited at 365 nm using a handy UV lamp. Figure 2D shows the UV–vis absorption spectra of (*S*_p_)-**11** in a mixed solution (1.0 × 10^–5^ M) of THF and H_2_O. The spectra were gradually red-shifted with increasing H_2_O volume. A broad band appeared as a tailing peak in the longer-wavelength region, when the H_2_O content was greater than 50 vol%, suggesting the formation of aggregates of (*S*_p_)-**11**. In Fig. 2E, the PL intensities are plotted against H_2_O vol%, indicating that the AIE of (*S*_p_)-**11** was correspondingly observed over 50 vol% of H_2_O.

Weak emission was detected around 445 nm, as shown in the inset of Fig. 2B, which can be observed in a series of X-shaped molecules^32,37,46,47^. These PL signals were not affected by the polarity change arose from the formation of aggregates; therefore, they were assigned to the general PL peaks of the X-shaped skeleton^32,37,46,47^. On the other hand, a clear blue-shift was observed for the strong emission peaks; for example, emission peak top in the THF/H_2_O (10/90) solution appeared at 609 nm, and that appeared at 771 nm in the THF/H_2_O (90/10) solution. It is suggested that the main emission of the carborane-containing π-conjugated molecules is caused by the intramolecular charge-transfer (vide infra) in the S_1_ state^7,9,10^. The aggregates are formed by hydrophobic effect in the H_2_O-concentrated solutions, and the inside of the aggregates becomes a low polar environment, leading to the blue-shift of the intramolecular CT emission peaks and the increase of the emission intensities owing to the immobility of the molecule^48,49^.

**Figure 3:**
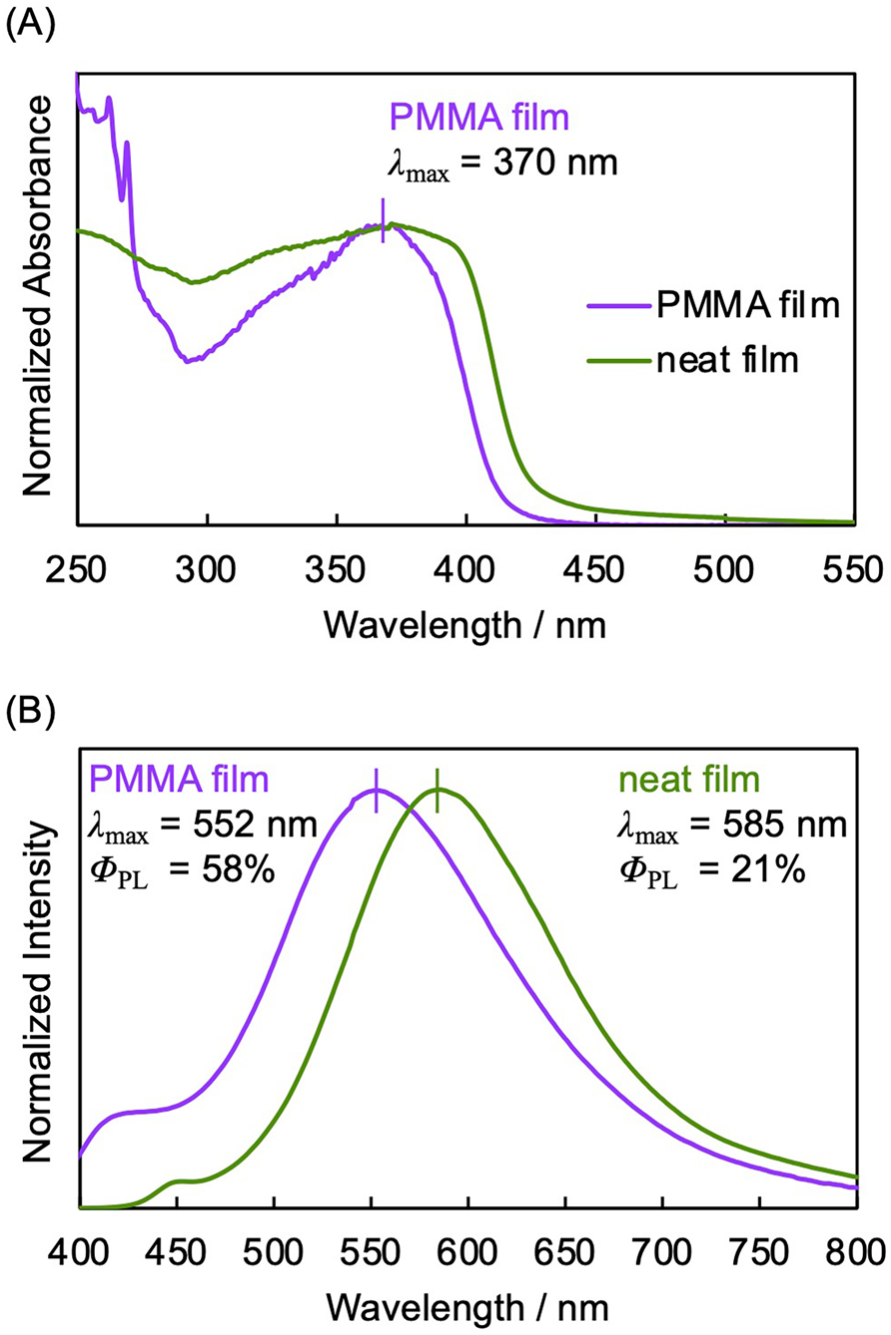
(**A**) UV–vis absorption spectra of the neat film of (*S*_p_)-**11** and the PMMA film containing 1 wt% of (*S*_p_)-**11**. (**B**) PL spectra of the neat film of (*S*_p_)-**11** and the PMMA film containing 1 wt% of (*S*_p_)-**11**, excited at 370 nm. The neat film was fabricated on a quartz plate by a drop-casting method using toluene solution; 0.5 mg of (*S*_p_)-**11** in toluene 5.0 mL. The PMMA film was fabricated on a quartz plate by a drop-casting method using toluene solution; 0.5 mg of (*S*_p_)-**11** and 49.5 mg of PMMA in toluene 5.0 mL. The obtained films were air-dried, and then, vacuum-dried.

Figure 3 shows the UV–vis absorption and PL spectra of the neat film of (*S*_p_)-**11** fabricated using the drop-casting method from the toluene solution containing 1 wt% of (*S*_p_)-**11** and a poly(methyl methacrylate) (PMMA) film containing 1 wt% of (*S*_p_)-**11**. The UV–vis absorption spectrum of the neat film reached a ceiling, exhibiting a red-shift attributed to the intermolecular interactions. The PL spectra of the neat film were also red-shifted compared to those of the (*S*_p_)-**11**-dispersed PMMA film. The calculated *Φ*_PL_s of the neat and PMMA films were 21% and 58%, respectively. In the PMMA film, (*S*_p_)-**11** was dispersed and isolated, resulting in a bright yellow AIE.

**Figure 4:**
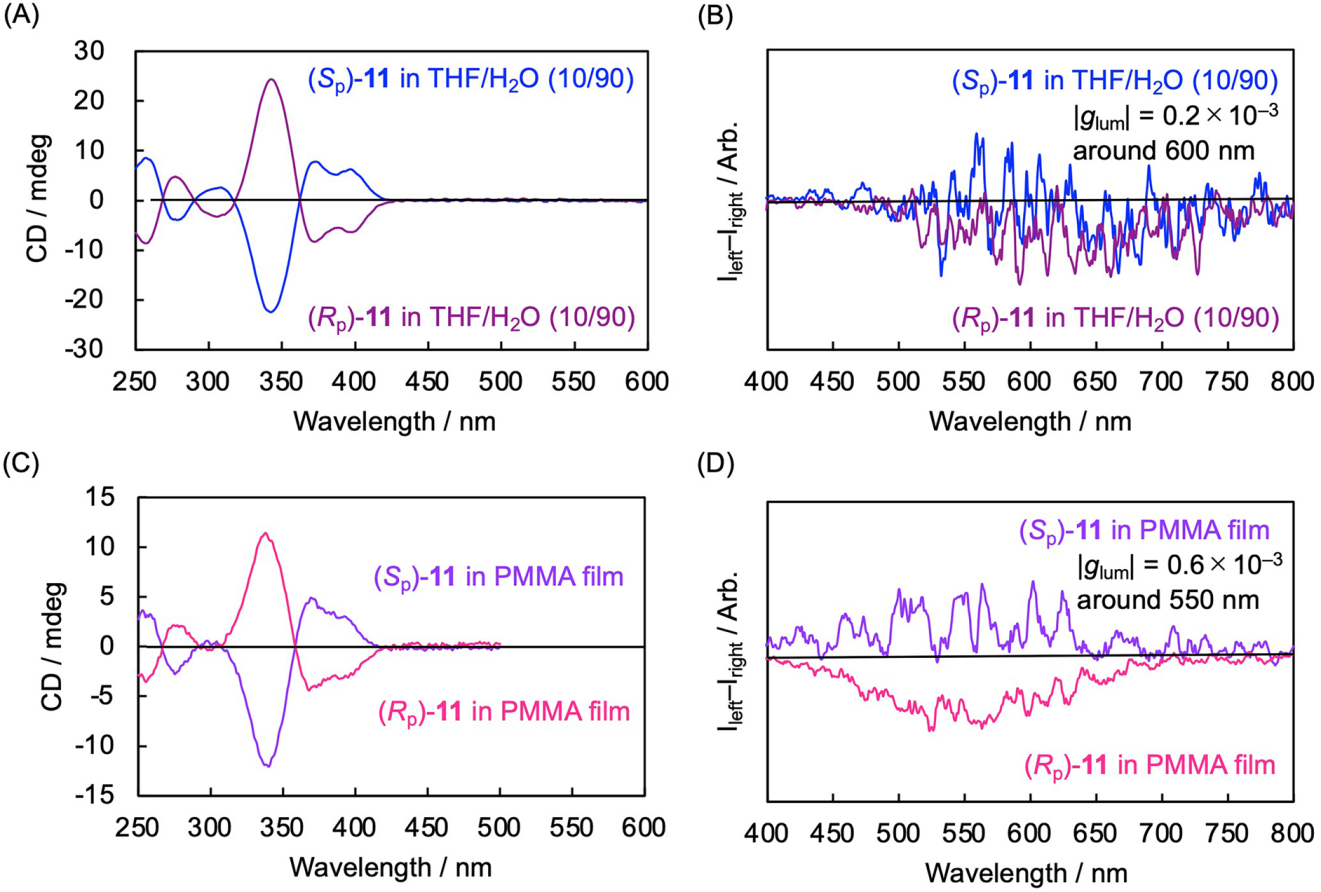
(**A**) CD spectra of (*S*_p_)-**11** in THF/H_2_O (10/90 vol/vol, 1.0 × 10^–5^ M). (**B**) CPL spectra of (*S*_p_)-**11** in THF/H_2_O (10/90 vol/vol, 1.0 × 10^5^ M), excited at 300 nm. (**C**) CD spectra of the PMMA film containing 1 wt% of (*S*_p_)-**11**. (**D**) CPL spectra of the PMMA film containing 1 wt% of (*S*_p_)-**11**, excited at 300 nm.

The CD and CPL properties of the aggregates of **11** in THF and H_2_O (H_2_O 90 vol%) and **11**-dispersed PMMA film and were investigated; their spectra are shown in Fig. 4. As shown in Fig. 4A, the mirror image CD spectra were observed for both the enantiomers of aggregates **11**, with the first Cotton effects of the (*S*_p_)- and (*R*_p_)-isomers being positive and negative, respectively. These signs are identical to those of a series of optically active X-shaped molecules reported previously^32,36,37,46^. However, their noisy CPL signals were barely detected (Fig. 4B), its absolute anisotropy factor (|*g*_lum_| value) was estimated to be 0.2 × 10^–3^ at the highest. The signs were the same as those of the first Cotton effects. In the aggregates, the molecules were π-stacked intermolecularly without ordered structures, namely, random higher-ordered structures, resulting in weak and noisy CPL spectra.

Figure 4C shows the CD spectra of the PMMA films including **11**, and the mirror image spectra were observed. The spectra and signs were similar to those of the aggregates (Fig. 4A). As shown in Fig. 4D, the CPL was clearly observed, and the signs were also identical to those of the first Cotton effects. The |*g*_lum_| value of the PMMA films containing **11** was calculated to be 0.6 × 10^–3^.

**Figure 5:**
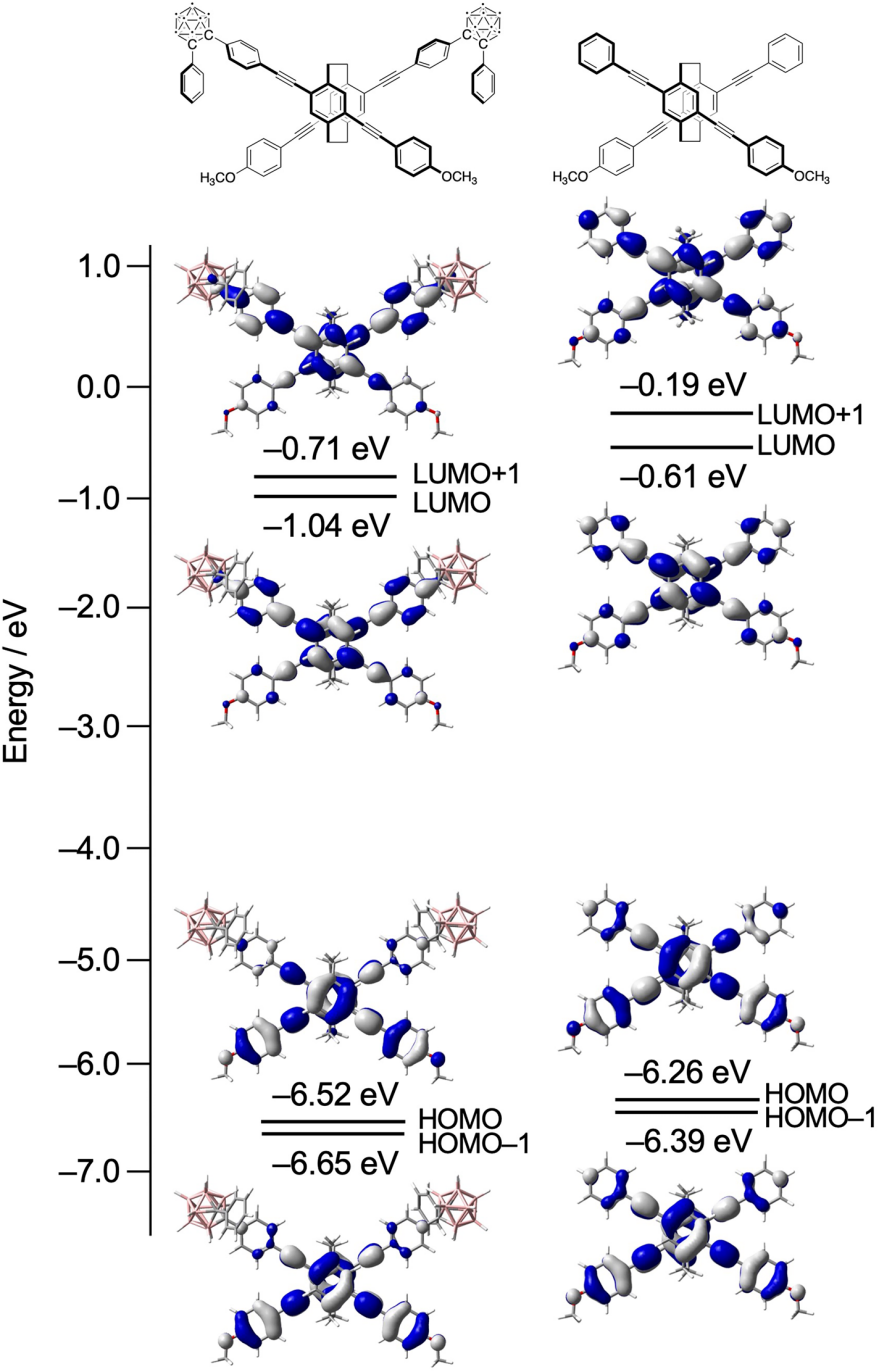
Molecular orbitals of the (*S*_p_)-**11** model and the corresponding X-shaped molecule in the ground states determined with TD-DFT calculations (TD-CAM-B3LYP/6-31G(d)).

The molecular orbitals of the model compound, in which the dodecyloxy groups of (*S*_p_)-**11** were replaced with methoxy groups, in the ground state were calculated using density functional theory (DFT) and compared with those of the X-shaped molecule prepared recently^46^, as shown in Fig. 5. Both molecules exhibited delocalized orbitals (highest occupied molecular orbital (HOMO), HOMO–1, lowest unoccupied molecular orbital (LUMO), and LUMO + 1) extending over the entire molecule via through-space interactions between the central benzene rings. The LUMO and LUMO + 1 in the (*S*_p_)-**11** model were primarily located on the benzene rings with carborane units, and the HOMO and HOMO–1 were primarily located on the methoxybenzene rings owing to the electron-withdrawing character of the carborane moiety. The carborane units decreased all the energy levels in the (*S*_p_)-**11** model, resulting in lower energy levels than those in the corresponding X-shaped molecule. The simulated CD spectrum of the (*S*_p_)-**11** model and parameters related to the transitions, which was simulated using time-dependent DFT (TD-DFT) calculations, are shown in Supplementary Fig. 30 and Supplementary Table 1 in the Supporting Information (SI). Transition from the HOMO to LUMO was allowed according to the oscillator strength, and the strong rotatory strength was estimated (Supplementary Table 1). The sign of the first Cotton effect observed in the (*S*_p_)-**11** model was positive, supporting the experimental results for (*S*_p_)-**11** (Fig. 4C).

**Figure 6:**
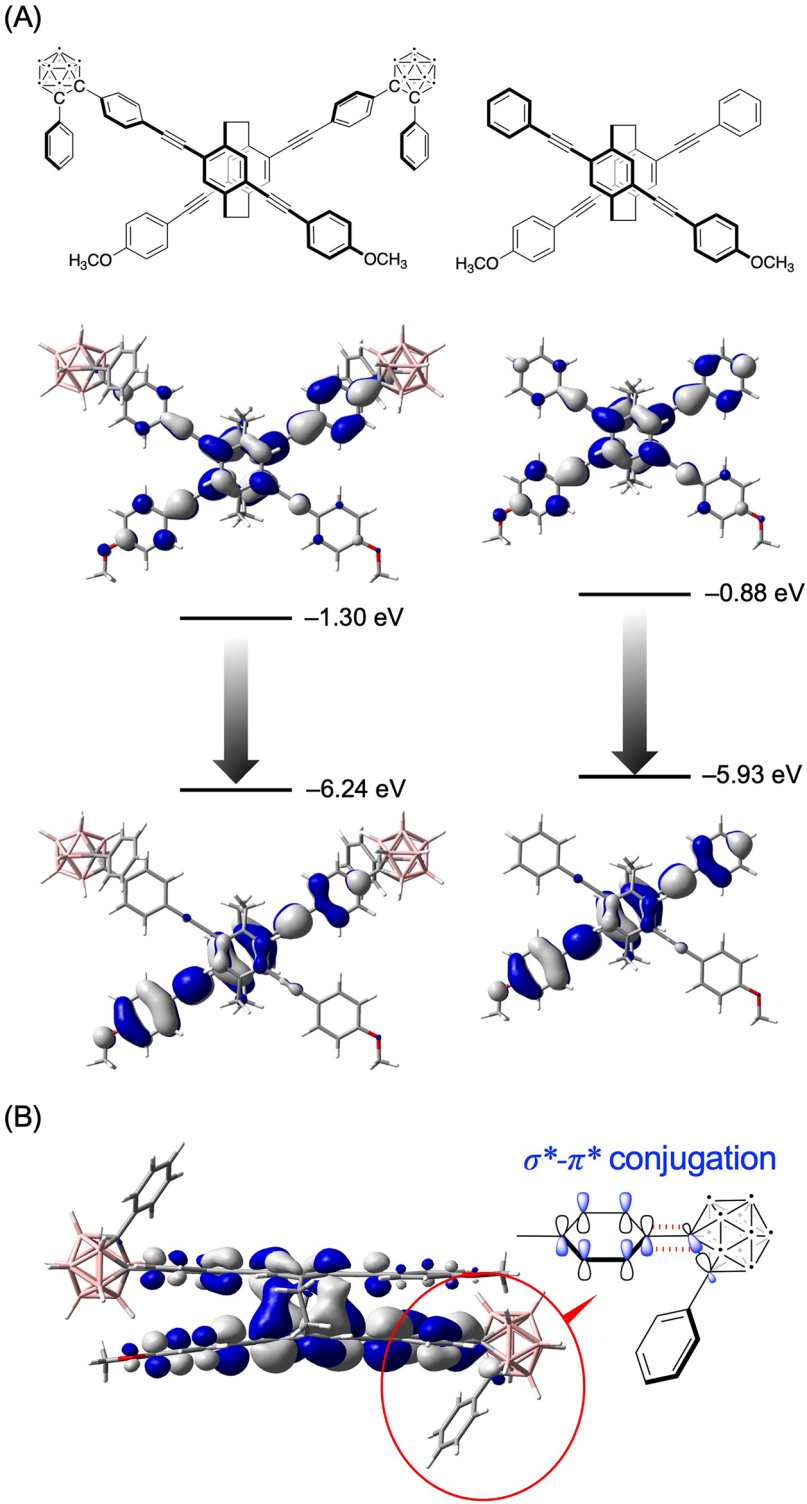
(**A**) Molecular orbitals of the (*S*_p_)-**11** model and the corresponding X-shaped molecule in the S_1_ states determined with TD-DFT calculations (TD-CAM-B3LYP/6-31G(d)). (**B**) Side view of the HOMO of the (*S*_p_)-**11** model in the S_1_ state, showing σ*-π* conjugation.

Figure 6A shows the LUMOs and HOMOs in the S_1_ states of the (*S*_p_)-**11** model, and Fig. 6B shows those of the corresponding X-shaped molecule^46^. Their orbitals were similar; the LUMOs were delocalized throughout the molecules, and the HOMOs were localized on one of the two π-electron systems. Figure 6B presents a side view of the LUMO in the S_1_ state of the (*S*_p_)-**11** model. The antibonding orbital of the C–C bond in the carborane unit was overlapped with a portion of the antibonding orbitals of the benzene ring to form σ*-π* conjugation^7,9,10^. The LUMO and HOMO in the in the S_1_ states of the (*S*_p_)-**11** model were slightly biased toward the carborane and methoxybenzene moieties, respectively, highlighting the CT emission of (*S*_p_)-**11**.

**Figure 7:**
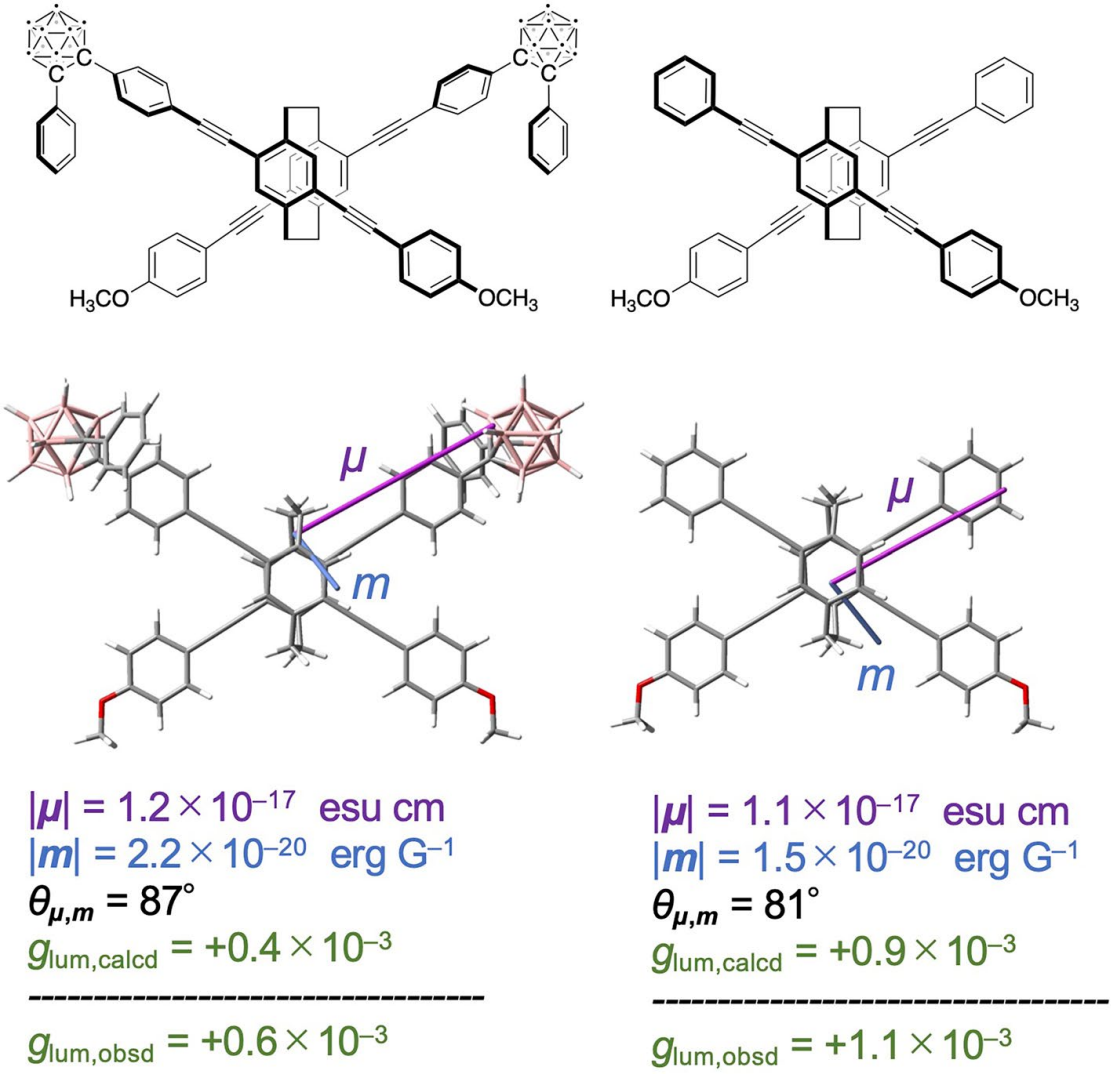
Simulation results for the transition dipole moments (***μ*** = electric transition dipole moment and ***m*** = magnetic transition dipole moment) of the (*S*_p_)-**11** model and the corresponding X-shaped molecule in the S_1_ state (TD-CAM-B3LYP/6-31G(d)//CAM-B3LYP/6-31G(d)).

Figure 7 shows the simulation results for the electric and magnetic transition dipole moments (***μ*** and ***m***, respectively) in the S_1_ states of the (*S*_p_)-**11** model and the corresponding X-shaped molecule^46^. The CPL *g*_lum,calcd_ value can be calculated using the following equation: *g*_lum,calcd_ = 4|***μ***||***m***|cos*θ*_*μ,m*_/(|***μ***|^2^ +|***m***|^2^), where *θ*_*μ,m*_ is the angle between ***μ*** and ***m***^29–31^. The ***μ***s in both molecules were extended along the long axis of one of the π-electron systems. The *θ*_*μ,m*_ of the (*S*_p_)-**11** model was closer to 90° than that of the corresponding X-shaped molecule, and the smaller *g*_lum,calcd_ value of + 0.4 × 10^–3^ was obtained. The observed *g*_lum,obsd_ values of (*S*_p_)-**11** and the X-shaped molecule were found to be + 0.6 × 10^–3^ and + 1.1 × 10^–3^, respectively. The sign and the order of the magnitude of the *g*_lum,calcd_ values were consistent with those of the observed *g*_lum,obsd_ values.

## Conclusion

Optically active π-stacked molecules comprising planar chiral [2.2]paracyclophane and *o*-carboranes were synthesized from enantiopure bis-(*para*)-pseudo-*meta*-tetrasubstituted [2.2]paracyclophanes. An X-shaped structure was constructed using the [2.2]paracyclophane unit, with *o*-carboranes located at the pseudo-*meta*-positions. Although the molecule did not exhibit emission in solution, it displayed emission in the form of aggregates (i.e. suspensions and films), exhibiting AIE properties. In particular, efficient emission was observed for molecules dispersed in the PMMA film. The PMMA film containing the molecule exhibited CPL properties with a moderate anisotropy factor of 0.6 × 10^–3^. This emission was derived from the CT emission from the carborane-containing benzene unit to the methoxybenzene unit. The CD and CPL behaviors were well-reproduced by simulations using TD-DFT calculations.

## Experimental section

### General

^1^H (500 MHz), ^13^C (125 MHz), and ^11^B (160 MHz) NMR spectra were recorded on a JEOL JNM ECZ-500R instrument. Samples were analyzed in CDCl_3_, and the chemical shift values were expressed relative to Me_4_Si for ^1^H and ^13^C NMR spectra and BF_3_·H_2_O spectra for ^11^B NMR for as internal standards. Analytical thin layer chromatography (TLC) was performed with silica gel 60 Merck F_254_ plates. Column chromatography was performed with Wakogel C-300 SiO_2_. Recyclable high-performance liquid chromatography (HPLC) was carried out on a YMC LC Forte/R. High-resolution mass (HRMS) spectra were obtained on a Bruker Daltonics microTOF II spectrometer (ESI and APCI) using sodium formate and tuning mix as internal standards. UV–vis spectra were recorded on a JASCO V-730 spectrophotometer, and samples were analyzed at room temperature. PL spectra were recorded on a JASCO FP-8500 spectrofluorometer, and samples were analyzed at room temperature. Absolute PL quantum efficiency was calculated on a JASCO FP8500 with an ILF-835 integrating sphere. Specific rotations ([*α*]^t^_D_) were measured with a HORIBA SEPA-500 polarimeter. CD spectra were recorded on a JASCO J-1500 spectropolarimeter with THF as a solvent at room temperature. CPL spectra were recorded on a JASCO CPL-300 with THF as a solvent at room temperature.

### Materials

Commercially available compounds used without purification are as follows: trimethylsilylacetylene, triisopropylsilylacetylene, decaborane (B_10_H_14_), *N*,*N*-dimethylaniline, *N*,*N*-dimethylformamide (DMF), Bu_4_NF (1.0 M THF), Pd_2_(dba)_3_, (*t*-Bu)_3_P·HBF_4_, dppf, CuI, KOH, K_2_CO_3_, KI, CH_2_Cl_2_, MeOH, THF (dehaydrated), toluene (dehaydrated), pyridine. Et_3_N was purchased and purified by distillation using KOH.

Compounds (*S*_p_)-**1**^43^, (*R*_p_)-**1**^43^, **5**^50^ and **9**^51^ were prepared according to the literature’s procedures.

### Synthesis of (*S*_p_)-2

A mixture of (*S*_p_)-**1** (660.2 mg, 0.99 mmol), Pd_2_(dba)_3_ (91.3 mg, 0.099 mmol), (*t*-Bu_3_)P·HBF_4_ (109.9 mg, 0.38 mmol), CuI (18.9 mg, 0.099 mmol), THF (10 mL), and Et_3_N (10 mL) was placed in a round-bottom flask equipped with magnetic stirring bar. After degassing the reaction mixture several times, trimethylsilylacetylene (1.44 mL, 11 mmol) was added to the mixture via a syringe. The reaction was carried out at reflux temperature for 1 day with stirring. After the reaction mixture was cooled to room temperature, precipitates were removed by filtration, and the solvent was removed with a rotary evaporator. The residue was purified by column chromatography on SiO_2_ (CHCl_3_/hexane = 1/5 v/v as an eluent) to afford (*S*_p_)-**2** (536.7 mg, 0.77 mmol, 77%) as a light yellow solid.

*R*_f_ = 0.35 (CHCl_3_/hexane = 1/5 v/v). ^1^H NMR (CDCl_3_, 500 MHz): δ 0.31 (s, 18H), 2.84–3.01 (m, 4H), 3.27–3.48 (m, 4H), 6.77 (s, 2H), 6.99 (s, 2H) ppm; ^13^C NMR (CDCl_3_, 125 MHz) δ = 29.9, 32.0, 101.1, 102.5, 118.8 (q, *J*_C–F_ = 325 Hz), 125.1, 126.2, 132.1, 137.0, 145.7, 148.0 ppm. HRMS (ESI +): m/z calcd. for C_28_H_30_F_6_O_6_S_2_Si_2_ [M + Na]^+^: 719.0819; found: 719.0834. [α]^25^_D_ =  + 39.46 (*c* 0.2, CHCl_3_).

The enantiomer (*R*_p_)-**2** was obtained in 90% yield by the same procedure. HRMS (ESI+): m/z calcd. for C_28_H_30_F_6_O_6_S_2_Si_2_ [M + Na]^+^: 719.0819; found: 719.0800. [α]^25^_D_ =  − 39.44 (*c* 0.2, CHCl_3_).

### Synthesis of (*S*_p_)-3

A mixture of (*S*_p_)-**2** (125.5 mg, 0.17 mmol), Pd_2_(dba)_3_ (16.09 mg, 0.017 mmol), dppf (9.75 mg, 0.017 mmol), CuI (3.34 mg, 0.017 mmol), THF (15 mL), and Et_3_N (15 mL) was placed in a round-bottom flask equipped with magnetic stirring bar. After degassing the reaction mixture several times, triisopropylsilylacetylene (0.52 mL, 1.7 mmol) was added to the mixture via a syringe. The reaction was carried out at reflux temperature for 2 days with stirring. After the reaction mixture was cooled to room temperature, precipitates were removed by filtration, and the solvent was removed with a rotary evaporator. The residue was purified by column chromatography on SiO_2_ (CHCl_3_/hexane = 1/9 v/v as an eluent) and by recyclable HPLC (CH_2_Cl_2_ as an eluent) to afford (*S*_p_)-**3** (118.7 mg, 0.15 mmol, 88%) as a light yellow oil.

*R*_f_ = 0.78 (CHCl_3_/hexane = 1/9 v/v). ^1^H NMR (CDCl_3_, 500 MHz): δ 0.29 (s, 18H), 1.19 (s, 42H), 2.90–2.99 (m, 4H), 3.37–3.45 (m, 4H), 6.98 (s, 2H), 7.01 (s, 2H) ppm; ^13^C NMR (CDCl_3_, 125 MHz) δ = 11.4, 18.6, 32.1, 95.5, 99.4, 104.5, 106.2, 124.9, 125.2, 134.2, 134.4, 142.1, 142.4 ppm. HRMS (ESI+): m/z calcd. for C_48_H_72_Si_4_ [M + Na]^+^: 786.4603; found: 786.4608. [α]^25^_D_ =  + 42.49 (*c* 0.1, CHCl_3_).

The enantiomer (*R*_p_)-**3** was obtained in 89% yield by the same procedure. HRMS (ESI): m/z calcd. for C_48_H_72_Si_4_ [M + Na]^+^: 786.4603; found: 786.4604. [α]^25^_D_ =  − 42.66 (*c* 0.1, CHCl_3_).

### Synthesis of (*S*_p_)-4

K_2_CO_3_ (142.5 mg, 1.03 mmol) was added to a suspension of (*S*_p_)-**3** (196.3 mg, 0.25 mmol) in MeOH (80 mL) and CH_2_Cl_2_ (10 mL). After the mixture was stirred for 17 h at room temperature, H_2_O was added to the reaction mixture. The organic layer was extracted with CHCl_3_ and washed with brine. The combined organic layer was dried over MgSO_4_. MgSO_4_ was removed by filtration, and the solvent was removed with a rotary evaporator. The residue was purified by column chromatography on SiO_2_ (CHCl_3_/hexane = 1/9 v/v as an eluent) to afford (*S*_p_)-**4** (116.8 mg, 0.18 mmol, 73%) as colorless needles.

*R*_f_ = 0.69 (CHCl_3_/hexane = 1/9 v/v). ^1^H NMR (CDCl_3_, 500 MHz): δ 1.19 (s, 42H), 2.94–3.01 (m, 4H), 3.31 (s, 2H), 3.38–3.47 (m, 4H), 7.03 (s, 2H), 7.09 (s, 2H) ppm; ^13^C NMR (CDCl_3_, 125 MHz) δ = 11.4, 18.7, 18.8, 31.9, 32.1, 81.8, 82.8, 95.9, 106.0, 123.9, 125.8 134.7 134.9, 142.0, 142.3 ppm. HRMS (ESI): m/z calcd. for C_42_H_56_Si_2_ [M + Na]^+^: 639.3813; found: 639.3805. [α]^25^_D_ =  + 52.57 (*c* 0.2, CHCl_3_).

The enantiomer (*R*_p_)-**4** was obtained in 75% yield by the same procedure. HRMS (ESI): m/z calcd. for C_42_H_56_Si_2_ [M + Na]^+^: 639.3813; found: 639.3785. [α]^25^_D_ =  − 52.28 (*c* 0.2, CHCl_3_).

### Synthesis of (*S*_p_)-6

A mixture of (*S*_p_)-**4** (32.0 mg, 0.051 mmol), **5** (44.26 mg, 0.11 mmol), Pd_2_(dba)_3_ (4.74 mg, 0.0051 mmol), dppf (2.87 mg, 0.0051 mmol), CuI (0.98 mg, 0.0051 mmol), THF (10 mL), and Et_3_N (10 mL) was placed in a round-bottom flask equipped with magnetic stirring bar. The reaction was carried out at reflux temperature for 2 days with stirring. After the reaction mixture was cooled to room temperature, precipitates were removed by filtration, and the solvent was removed with a rotary evaporator. The residue was purified by column chromatography on SiO_2_ (CHCl_3_/hexane = 1/9 v/v as an eluent) and by recyclable HPLC (CH_2_Cl_2_ as an eluent) to afford (*S*_p_)-**6** (36.5 mg, 0.032 mmol, quant.) as a light yellow oil.

*R*_f_ = 0.20 (CHCl_3_/hexane = 1/9 v/v). ^1^H NMR (CDCl_3_, 500 MHz): δ 0.88 (t, *J* = 7.0 Hz, 6H), 1.19 (s, 42H), 1.27–1.50 (36H), 1.80 (m, 4H), 3.00–3.04 (m, 4H), 3.44–3.53 (m, 4H), 3.99 (t, *J* = 6.5 Hz, 4H), 6.89 (d, *J* = 8.6 Hz, 4H), 7.07 (s, 2H), 7.09 (s, 2H), 7.48 (d, *J* = 8.0 Hz, 4H); ^13^C NMR (CDCl_3_, 125 MHz) δ = 11.5, 14.1, 18.8, 22.7, 26.0, 29.2, 29.4, 29.6, 31.9, 68.1, 87.7, 94.7, 95.2, 106.6, 114.4, 114.6, 115.4, 124.7, 125.5, 132.9, 130.0, 133.8, 134.0, 134.9, 135.1, 141.6, 142.2, 159.2 ppm. HRMS (ESI): m/z calcd. for C_78_H_112_O_2_Si_2_ [M + Na]^+^: 1159.8093; found: 1159.8078. [α]^25^_D_ =  + 331.18 (*c* 0.1, CHCl_3_).

The enantiomer (*R*_p_)-**6** was obtained in 88% yield by the same procedure. HRMS (ESI): m/z calcd. for C_78_H_112_O_2_Si_2_ [M + Na]^+^: 1159.8093; found: 1159.8136. [α]^25^_D_ =  − 331.23 (*c* 0.1, CHCl_3_).

### Synthesis of (*S*_p_)-7

(*S*_p_)-**6** (87.0 mg, 0.076 mmol) was dissolved in THF (15 mL), followed by the addition of Bu_4_NF (1.0 M THF solution, 1.0 mL) via a syringe. The reaction was carried out at room temperature for 10 min, H_2_O was added to the reaction mixture. The organic layer was extracted three times with CHCl_3_ and washed with brine, and dried over MgSO_4_. MgSO_4_ was removed by filtration, and the solvent was removed by a rotary evaporator. The residue was purified by column chromatography on SiO_2_ (CHCl_3_/hexane = 2/3 v/v as an eluent) and by recyclable HPLC (CH_2_Cl_2_ as an eluent) to afford (*S*_p_)-**7** (40.0 mg, 0.048 mmol, quant.) as a light yellow oil.

*R*_f_ = 0.55 (CHCl_3_/hexane = 2/3 v/v). ^1^H NMR (CDCl_3_, 500 MHz): δ 0.88 (t, *J* = 7.0 Hz, 6H), 1.27–1.49 (36H), 1.80 (m, 4H), 3.00–3.05 (m, 4H), 3.37 (s, 2H), 3.43–3.50 (m, 4H), 3.99 (t, *J* = 7.0 Hz, 4H), 6.90 (d, *J* = 8.6 Hz, 4H), 7.03 (s, 2H), 7.11 (s, 2H), 7.50 (d, *J* = 8.6 Hz, 4H) ppm; ^13^C NMR (CDCl_3_, 125 MHz) δ = 14.1, 22.7, 26.0, 29.1, 29.3, 29.5, 29.6, 31.9, 32.0, 32.5, 68.1, 81.5, 83.1, 87.4, 94.8, 114.6, 115.3, 123.2, 126.1, 132.9, 134.1, 134.9, 141.6, 142.5, 159.3 ppm. HRMS (ESI): m/z calcd. for C_60_H_72_O_2_ [M + Na]^+^: 847.5425; found: 847.5389. [α]^25^_D_ =  + 55.51 (*c* 0.1, CHCl_3_).

The enantiomer (*R*_p_)-**7** was obtained quantitatively by the same procedure. HRMS (ESI): m/z calcd. for C_60_H_72_O_2_ [M + Na]^+^: 847.5425; found: 847.5416. [α]^25^_D_ =  − 55.29 (*c* 0.05, CHCl_3_).

### Synthesis of 10

The mixture of compound **9** (914.2 mg, 3.01 mmol) and decaborane **8** (423.5 mg, 3.46 mmol) was dissolved in dry toluene (15 mL) at room temperature. *N*,*N*-Dimethylaniline (0.64 mL, 5.04 mmol) was added, and the mixture was refluxed for 24 h. After cooling to room temperature, solvent was separated from the solid and evaporated. The residue was purified by column chromatography on SiO_2_ (hexane as an eluent). Recrystallization from CHCl_3_ and MeOH to provide compound **10** (862.9 mg, 2.03 mmol, 68%) as a colorless crystal.

*R*_f_ = 0.28 (hexane). ^1^H NMR (CDCl_3_, 500 MHz): δ 1.90–3.71 (br, 10H), 7.11 (d, *J* = 8.6 Hz, 2H), 7.16 (t, *J* = 8.0, 2H), 7.27 (d, *J* = 7.0 Hz, 1H), 7.41 (d, *J* = 7.5 Hz, 2H), 7.46 (d, *J* = 8.6 Hz, 2H); ^13^C NMR (CDCl_3_, 125 MHz) δ = 84.1, 85.1, 97.1 128.4, 130.3, 130.4, 130.5, 132.0. 137.4 ppm; ^11^B NMR (CDCl_3_, 160 MHz) δ = 3.56, 2.62, − 3.20, − 4.33, − 5.36, − 6.61 ppm. HRMS (ESI): m/z calcd. for C_14_H_19_B_10_I [M + Cl]^–^: 459.1161; found: 459.1159.

### Synthesis of (*S*_p_)-11

A mixture of (*S*_p_)-**7** (40.0 mg, 0.048 mmol), **10** (49.23 mg, 0.116 mmol), Pd_2_(dba)_3_ (4.43 mg, 0.0048 mmol), dppf (5.37 mg, 0.0096 mmol), CuI (1.84 mg, 0.0096 mmol), THF (7.5 mL), and Et_3_N (7.5 mL) was placed in a round-bottom flask equipped with magnetic stirring bar. The reaction was carried out at reflux temperature for 2 days with stirring. After the reaction mixture was cooled to room temperature, precipitates were removed by filtration, and the solvent was removed with a rotary evaporator. The residue was purified by column chromatography on SiO_2_ (CHCl_3_/hexane = 1/4 v/v as an eluent) and by recyclable HPLC (CH_2_Cl_2_ as an eluent) to afford (*S*_p_)-**11** (27.9 mg, 0.019 mmol, 81%) as a yellow solid.

*R*_f_ = 0.28 (CHCl_3_/hexane = 1/4 v/v). ^1^H NMR (CDCl_3_, 500 MHz): δ 0.88 (t, *J* = 6.5 Hz, 6H), 1.27–1.49 (36H), 1.83 (m, 4H), 1.90–2.88 (br, 20H), 2.92–3.06 (m, 4H), 3.38–3.50 (m, 4H), 4.02 (t, *J* = 6.5 Hz, 4H), 6.90 (d, *J* = 8.0 Hz, 4H), 6.95 (s, 2H), 7.08 (s, 2H), 7.17 (t, *J* = 8.0, 4H), 7.27–7.31 (m, 6H), 7.42–7.47 (m, 12H); ^13^C NMR (CDCl_3_, 125 MHz) δ = 14.1, 22.7, 26.0, 29.2, 29.3, 29.4, 29.6, 31.9, 32.5, 68.2, 84.5, 85.4, 87.6, 91.8, 92.7, 95.2, 114.6, 115.2, 123.8, 125.6, 126.1, 128.4, 130.3, 130.5, 130.6, 131.1, 132.9, 134.4, 134.6, 141.7, 159.4 ppm; ^11^B NMR (CDCl_3_, 160 MHz) δ = 2.7, –5.0 ppm. HRMS (ESI): m/z calcd. for C_88_H_108_B_20_O_2_ [M + H]^+^: 1418.0422; found: 1418.0374. [α]^25^_D_ =  + 250.48 (*c* 0.1, CHCl_3_).

The enantiomer (*R*_p_)-**11** was obtained in 92% yield by the same procedure. HRMS (ESI): m/z calcd. for C_88_H_108_B_20_O_2_ [M + H]^+^: 1418.0422; found: 1418.0386. [α]^25^_D_ =  − 250.22 (*c* 0.1, CHCl_3_).

### Supplementary Information

Supplementary Information 1.

Supplementary Information 2.

## Data Availability

The datasets used and/or analyzed during the current study available from the corresponding author on reasonable request.
