# Supplementary material for: Aggregation-induced emission from optically active X-shaped molecules based on planar chiral [2.2]paracyclophane
Source: Sci Rep. 2023 Dec 19;13:22647. doi: 10.1038/s41598-023-49120-2 (PMC10730888; doi:10.1038/s41598-023-49120-2)
Supplement: Supplementary file 1 — Supplementary Information 1. [file 41598_2023_49120_MOESM1_ESM.pdf]

Supporting Information

Aggregation-induced emission from optically active X-shaped molecules based on planar chiral [2.2]paracyclophane

Keishi Jikuhara, Ryo Inoue, Yasuhiro Morisaki*

Department of Applied Chemistry for Environment, School of Biological and Environmental Sciences, Kwansei Gakuin University
1 Gakuen Uegahara, Sanda, Hyogo 669-1330, Japan
E-mail: ymo@kwansei.ac.jp (Yasuhiro Morisaki)

Computational methods

DFT and TD-DFT calculations[1–5] were carried out for isolated molecules by using the Gaussian 16 program package[6], with the 6-31G(d)[7–9] basis set for C, H, and O atoms. The initial molecular geometry for ground state was obtained by combining crystal structures of *o*-carborane derivative (CCDC-1037359)[10] and X-shaped [2.2]paracyclophane derivative (CCDC-2184874).[11] Optimized geometries and their molecular orbitals in the ground and S_1 states were determined by DFT and TD-DFT calculations with the CAM-B3LYP[12] functional. Cartesian coordinates of all optimized structures are given in Tables S2–S5.

1) M. E. Casida, C. Jamorski, K. C. Casida, D. R. Salahub, *J. Chem. Phys.* **1998**, *108*, 4439–4449.
2) R. E. Stratmann, G. E. Scuseria, M. J. Frisch, *J. Chem. Phys.* **1998**, *109*, 8218–8224.
3) R. Bauernschmitt, R. Ahlrichs, *Chem. Phys. Lett.* **1996**, 256, 454–464.
4) C. Adamo, D. Jacquemin, *Chem. Soc. Rev.* **2013**, *42*, 845–856.
5) C. Adamo, T. Le Bahers, M. Savarese, L. Wilbraham, G. García, R. Fukuda, M. Ehara, N. Rega, I. Ciofini, *Coord. Chem. Rev.* **2015**, *304–305*, 166–178.
6) Gaussian 16, Revision B.01, M. J. Frisch, G. W. Trucks, H. B. Schlegel, G. E. Scuseria, M. A. Robb, J. R. Cheeseman, G. Scalmani, V. Barone, G. A. Petersson, H. Nakatsuji, X. Li, M. Caricato, A. V. Marenich, J. Bloino, B. G. Janesko, R. Gomperts, B. Mennucci, H. P. Hratchian, J. V. Ortiz, A. F. Izmaylov, J. L. Sonnenberg, D. Williams-Young, F. Ding, F. Lipparini, F. Egidi, J. Goings, B. Peng, A. Petrone, T. Henderson, D. Ranasinghe, V. G. Zakrzewski, J. Gao, N. Rega, G. Zheng, W. Liang, M. Hada, M. Ehara, K. Toyota, R. Fukuda, J. Hasegawa, M. Ishida, T. Nakajima, Y. Honda, O. Kitao, H. Nakai, T. Vreven, K. Throssell, J. A. Montgomery, Jr., J. E. Peralta, F. Ogliaro, M. J. Bearpark, J. J. Heyd, E. N. Brothers, K. N. Kudin, V. N. Staroverov, T. A. Keith, R. Kobayashi, J. Normand, K. Raghavachari, A. P. Rendell, J. C. Burant, S. S. Iyengar, J. Tomasi, M. Cossi, J. M. Millam, M. Klene, C. Adamo, R. Cammi, J. W. Ochterski, R. L. Martin, K. Morokuma, O. Farkas, J. B. Foresman, and D. J. Fox, Gaussian, Inc., Wallingford CT, 2016.
7) M. M. Francl, W. J. Pietro, W. J. Hehre, J. S. Binkley, M. S. Gordon, D. J. DeFrees, J. A. Pople, *J. Chem. Phys.* **1982**, *77*, 3654–3665.
8) P. C. Hariharan, J. A. Pople, *Theor. Chim. Acta* **1973**, *28*, 213–222.
9) T. Clark, J. Chandrasekhar, G. W. Spitznagel, P. V. R. Schleyer, *J. Comput. Chem.* **1983**, *4*, 294–0.
10) H. Naito, Y. Morisaki, Y. Chujo, *Angew. Chem. Int. Ed.* **2015**, *54*, 5084–5087.
11) O. Oki, H. Yamagishi, Y. Morisaki, R. Inoue, K. Ogawa, N. Miki, Y. Norikane, H. Sato, Y. Yamamoto, *Science* **2022**, *377*, 673–678.
12) T. Yanai, D. Tew, N. Handy, *Chem. Phys. Lett.* **2004**, *393*, 51–57.

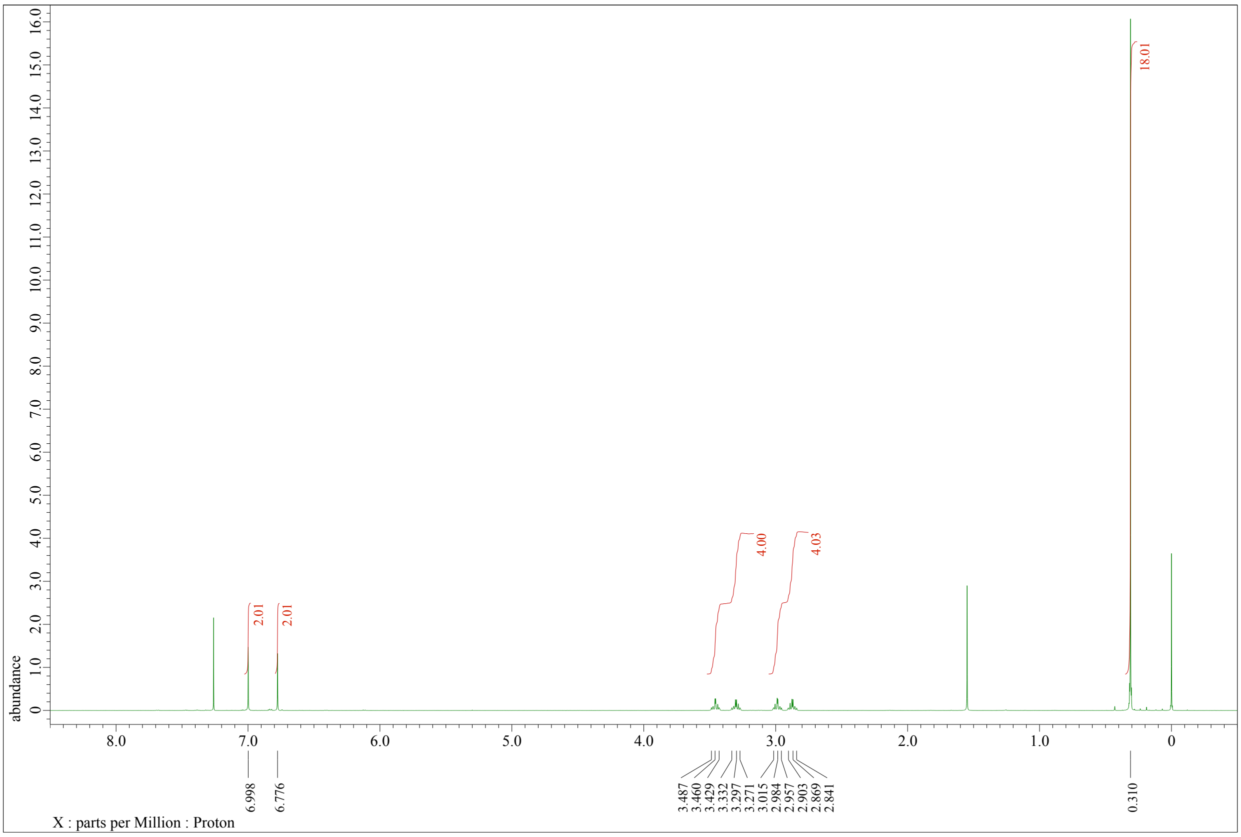

Supplementary Figure 1. ^{1}H NMR spectrum of (S_p)-**2** in $CDCl_3$.

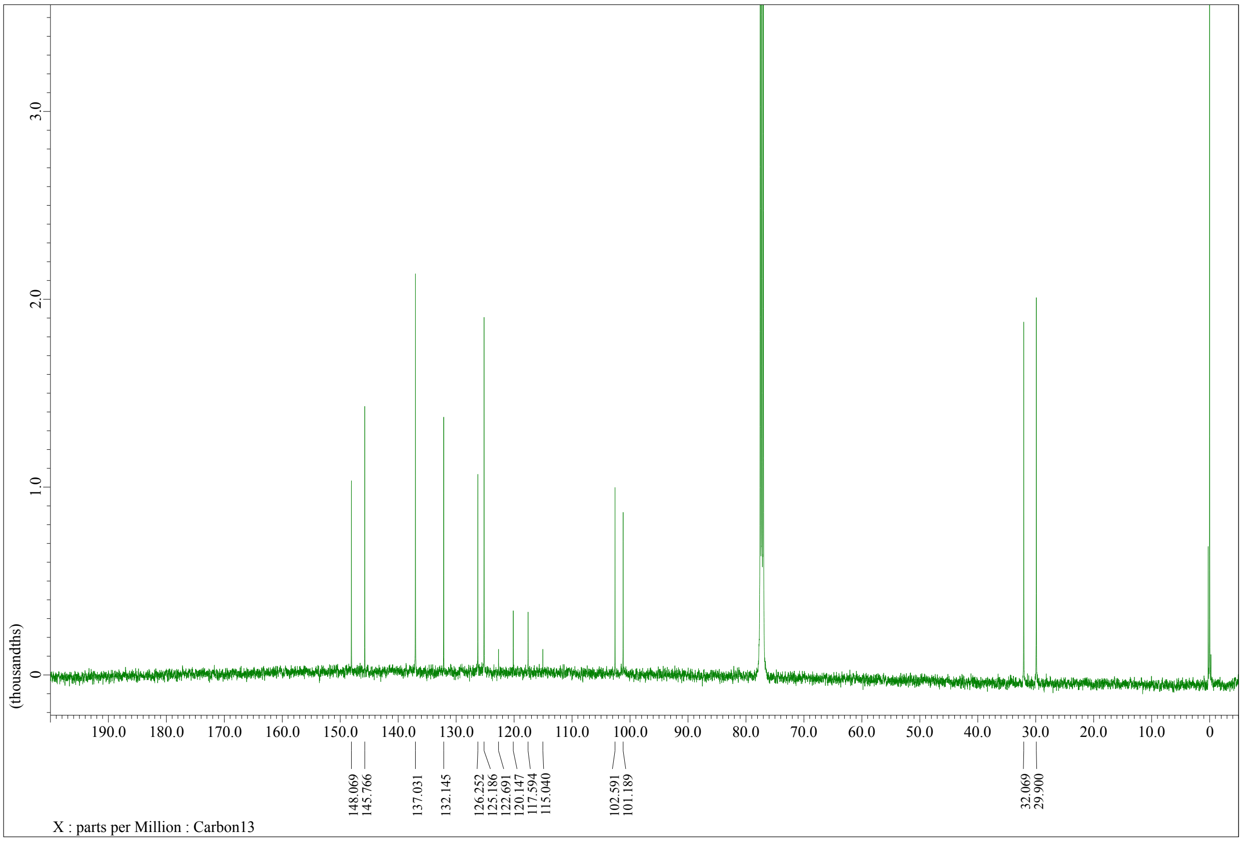

Supplementary Figure 2. ^{13}C NMR spectrum of (S_p)-**2** in $CDCl_3$.

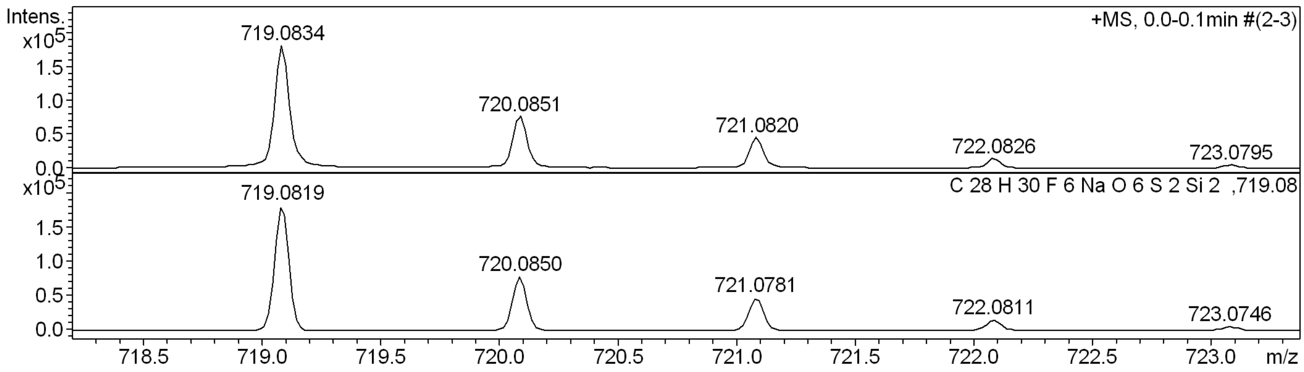

Supplementary Figure 3. Mass spectra (ESI) and data of (S_p)-**2**. Top: experimental spectrum and bottom: theoretical spectrum.

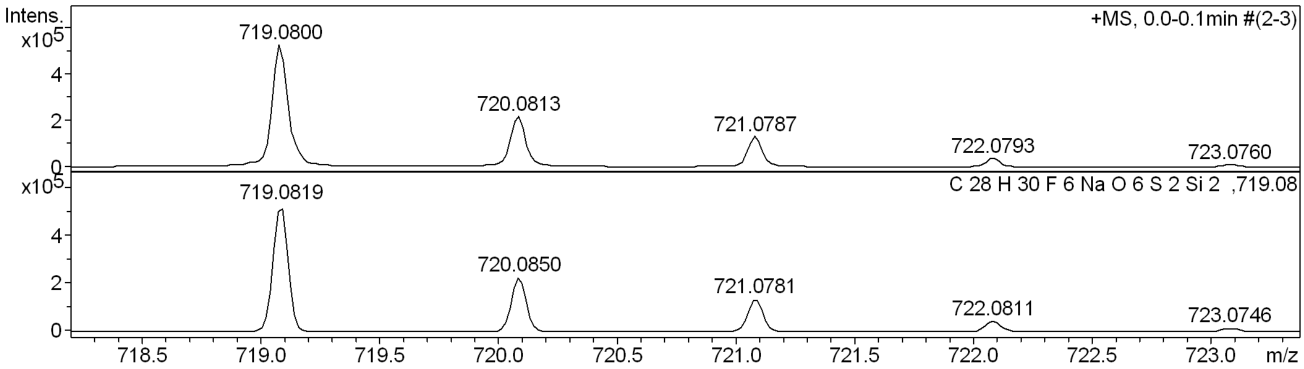

Supplementary Figure 4. Mass spectra (ESI) and data of (R_p)-**2**. Top: experimental spectrum and bottom: theoretical spectrum.

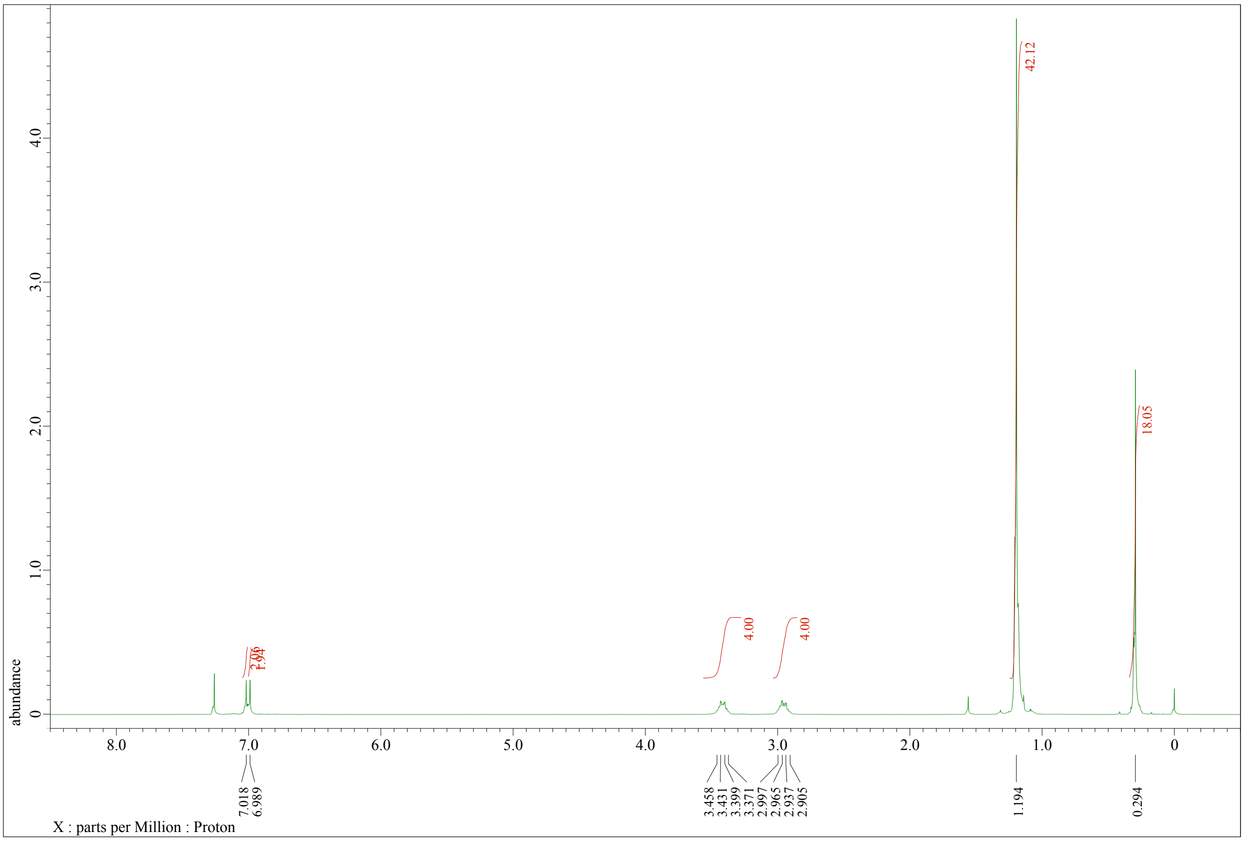

Supplementary Figure 5. ^{1}H NMR spectrum of (S_p)-**3** in $CDCl_3$.

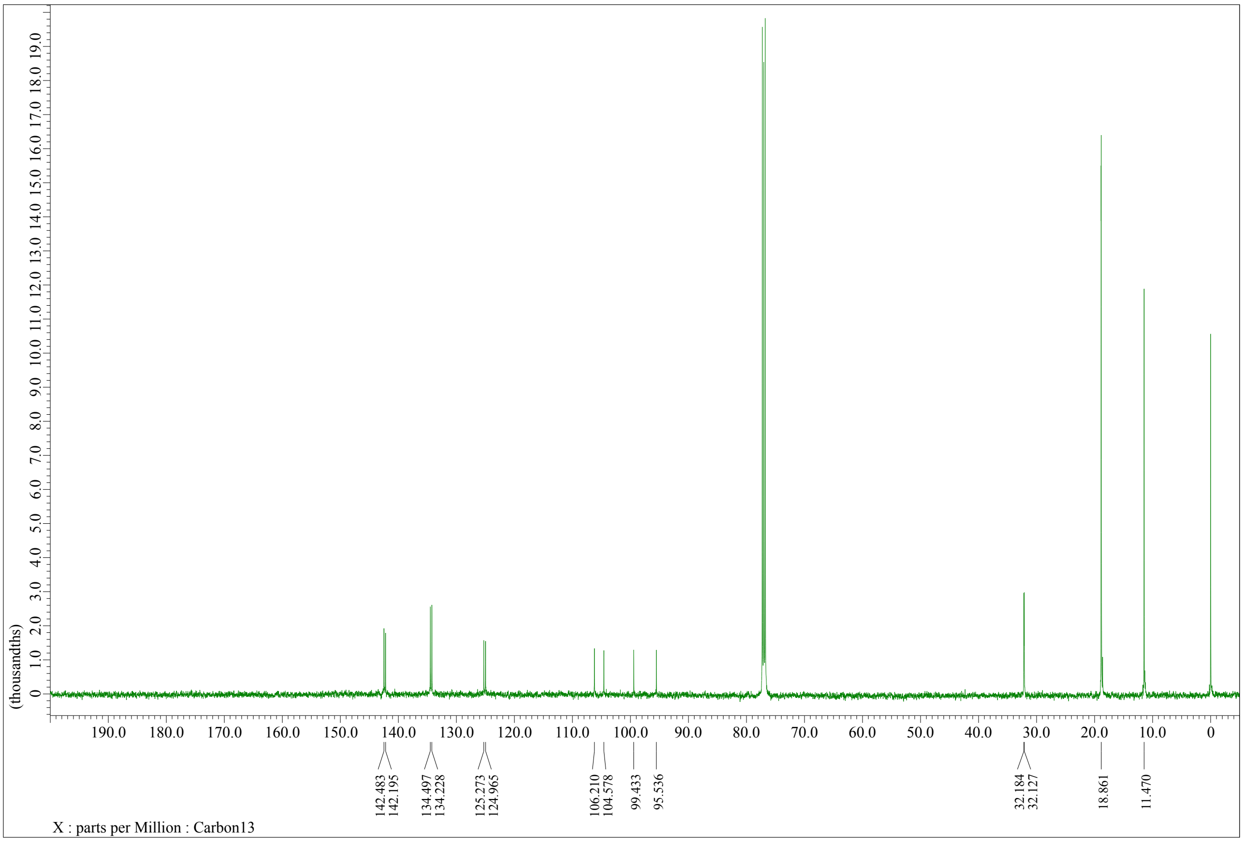

Supplementary Figure 6. ^{13}C NMR spectrum of (S_p)-**3** in $CDCl_3$.

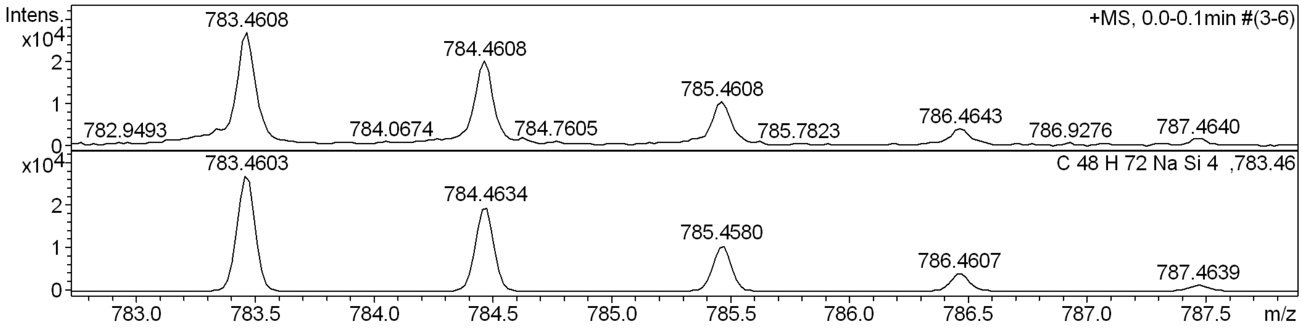

Supplementary Figure 7. Mass spectra (ESI) and data of (S_p)-**3**. Top: experimental spectrum and bottom: theoretical spectrum.

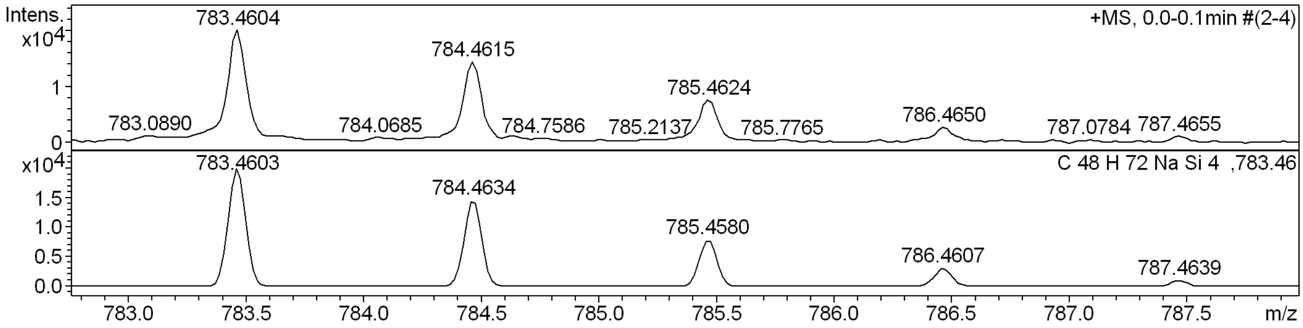

Supplementary Figure 8. Mass spectra (ESI) and data of (R_p)-**3**. Top: experimental spectrum and bottom: theoretical spectrum.

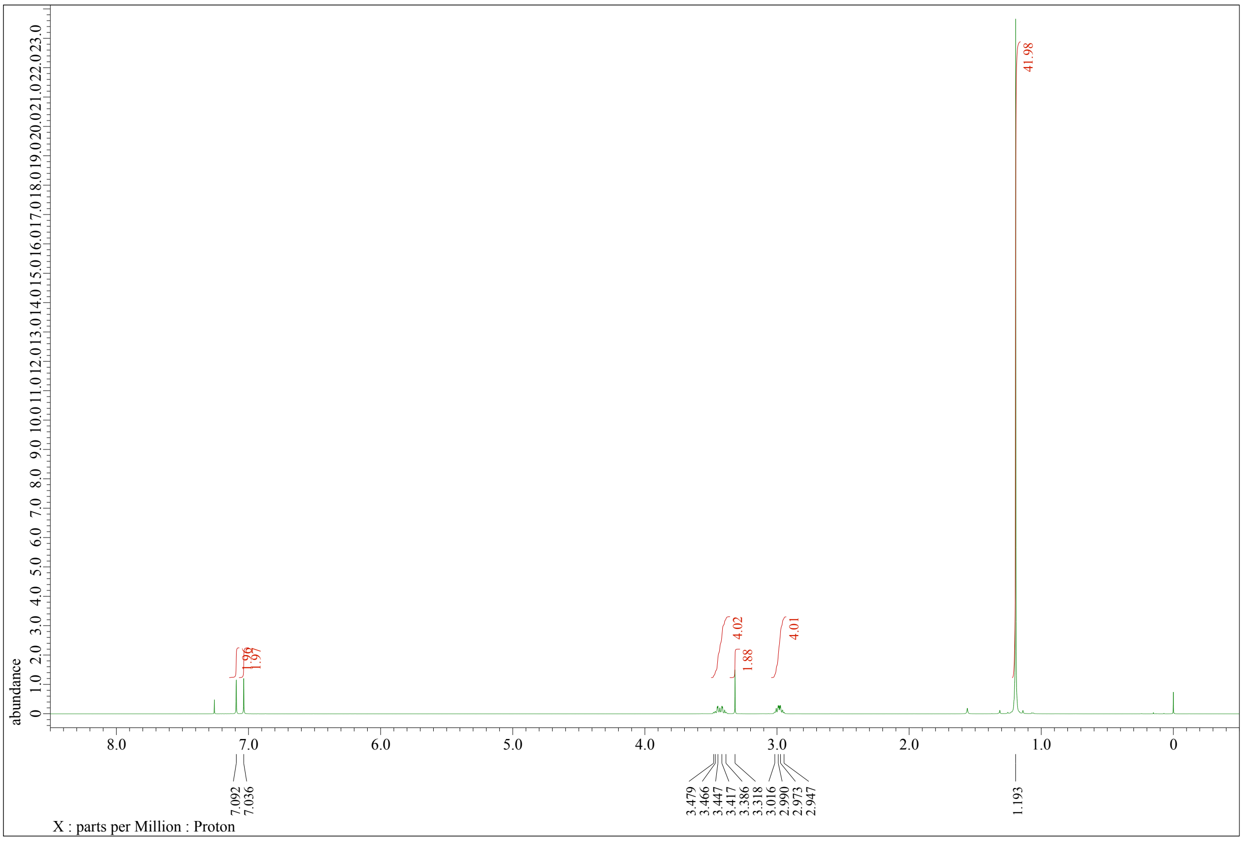

Supplementary Figure 9. ^{1}H NMR spectrum of (S_p)-**4** in $CDCl_3$.

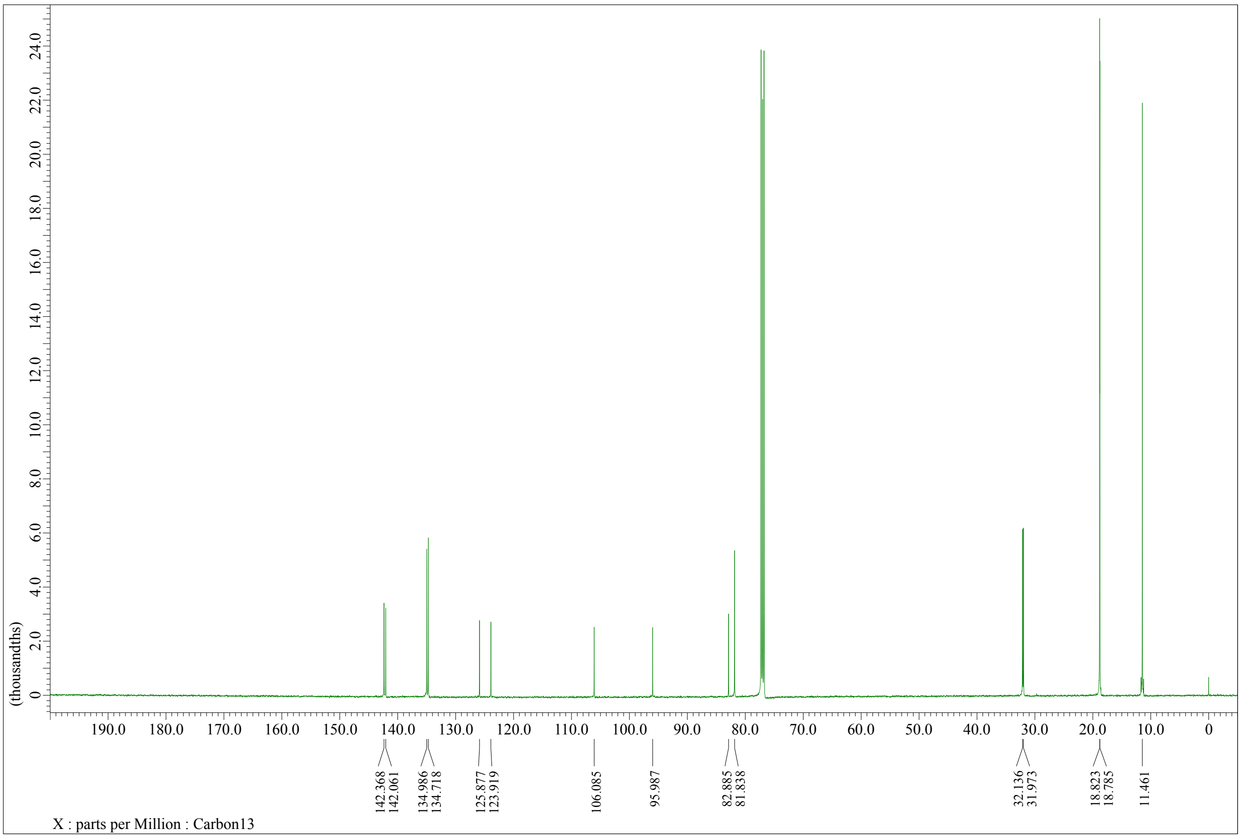

Supplementary Figure 10. ^{13}C NMR spectrum of (S_p)-**4** in $CDCl_3$.

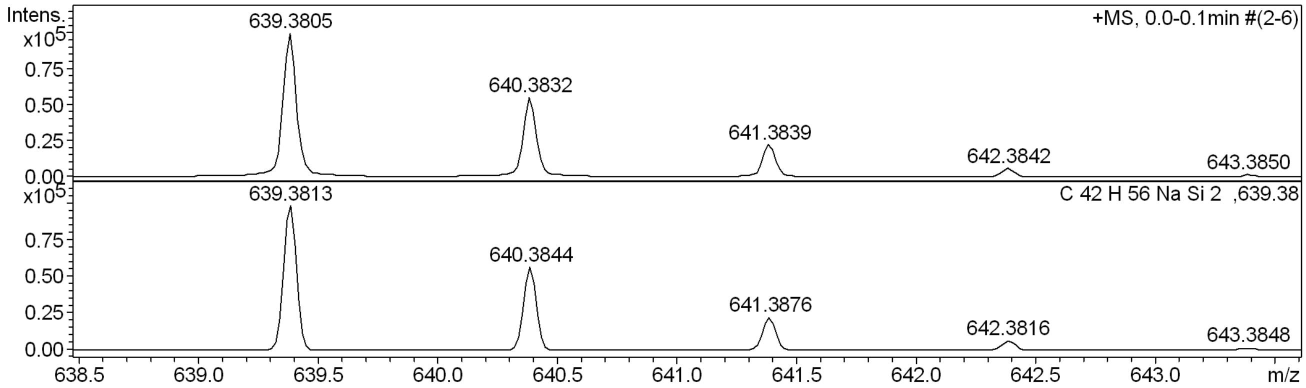

Supplementary Figure 11. Mass spectra (ESI) and data of (S_p)-**4**. Top: experimental spectrum and bottom: theoretical spectrum.

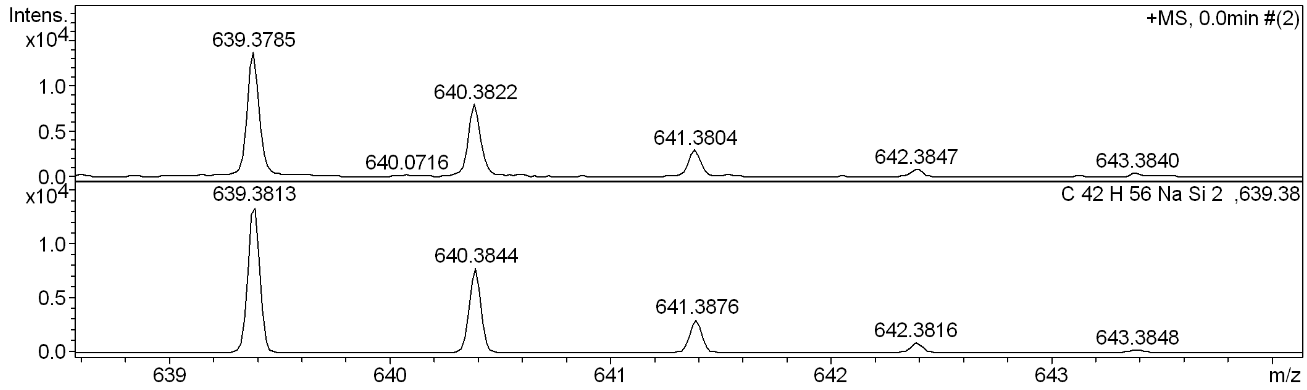

Supplementary Figure 12. Mass spectra (ESI) and data of (R_p)-**4**. Top: experimental spectrum and bottom: theoretical spectrum.

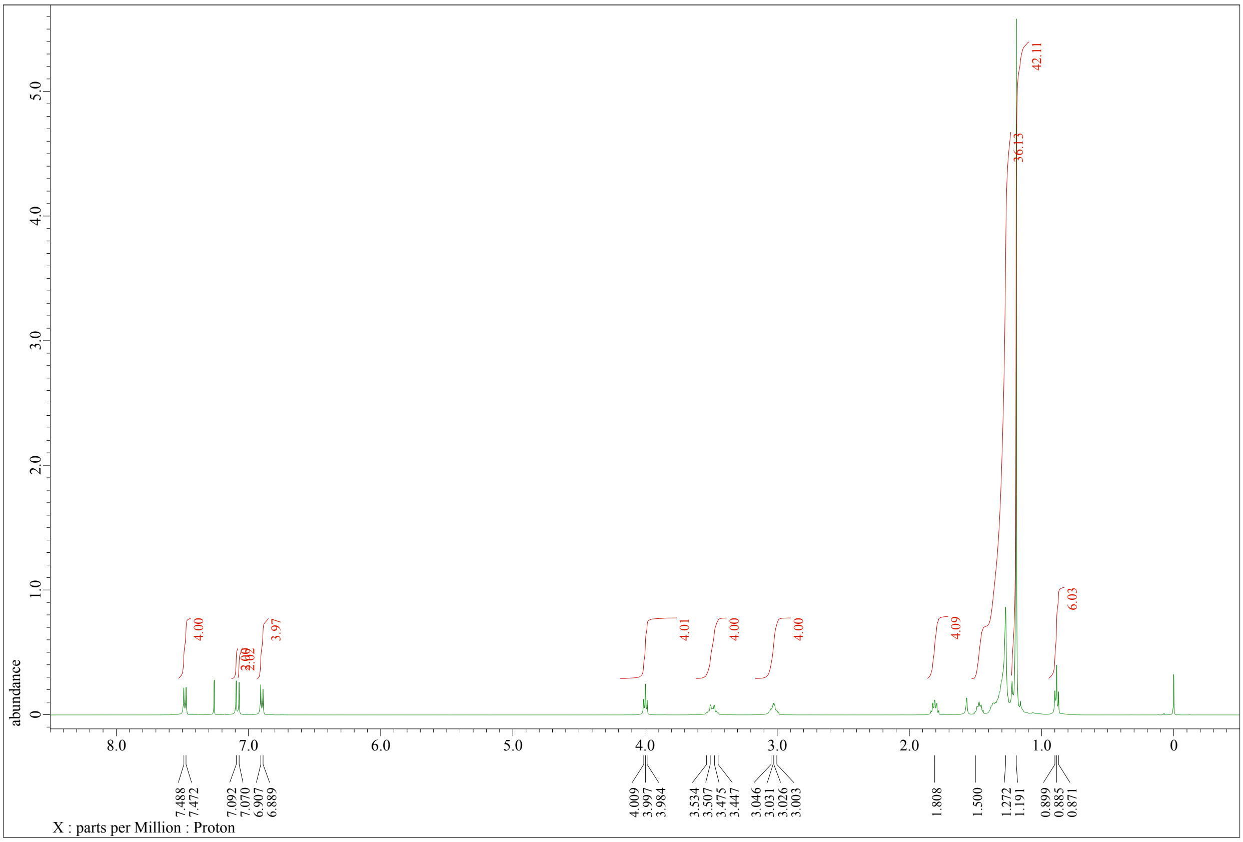

Supplementary Figure 13. ^{1}H NMR spectrum of (S_p)-**6** in $CDCl_3$.

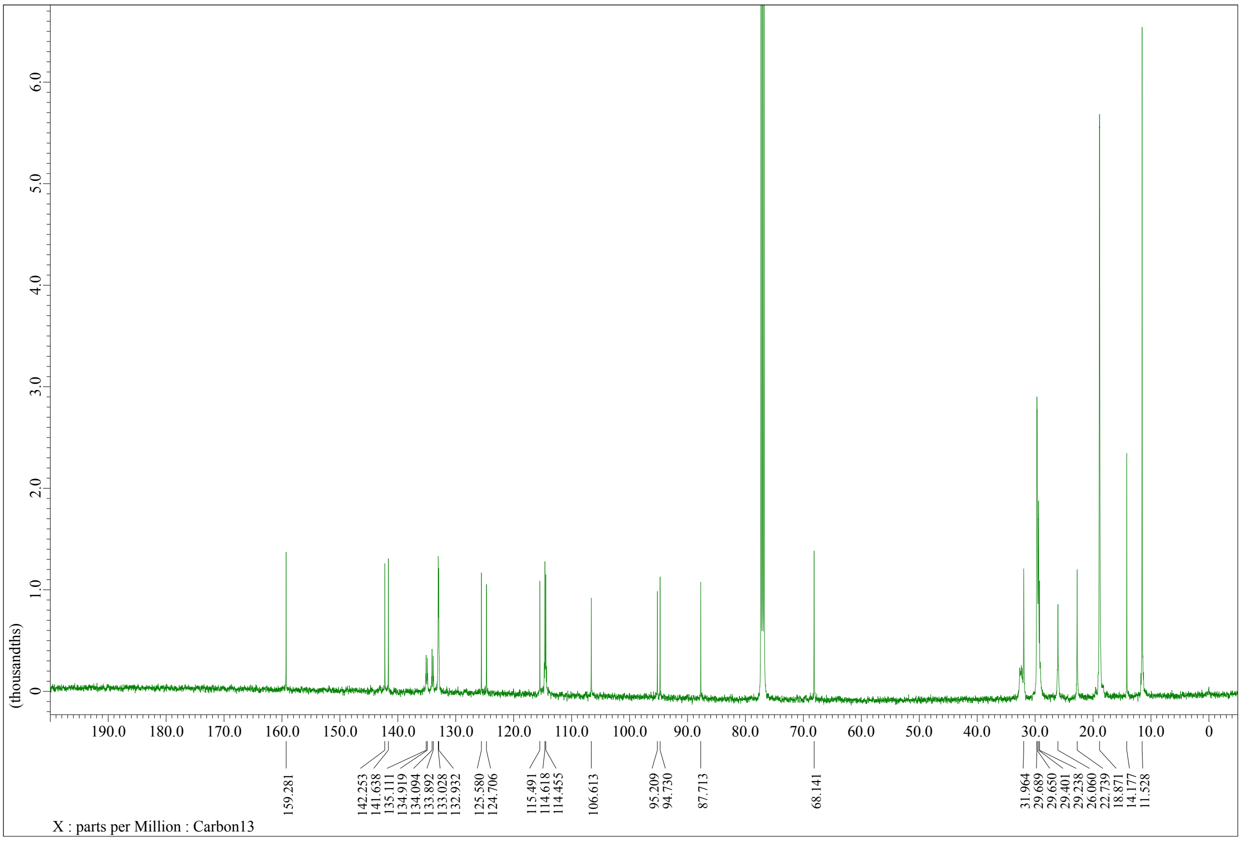

Supplementary Figure 14. ^{13}C NMR spectrum of (S_p)-**6** in $CDCl_3$.

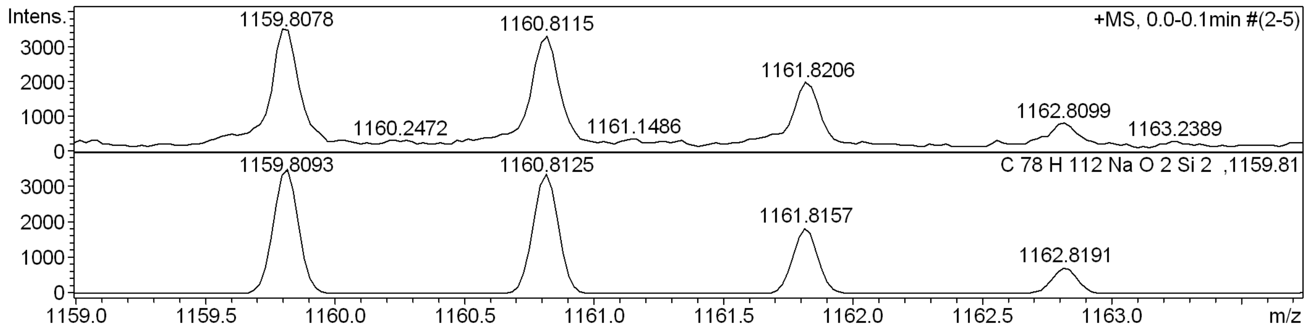

Supplementary Figure 15. Mass spectra (ESI) and data of (S_p)-**6**. Top: experimental spectrum and bottom: theoretical spectrum.

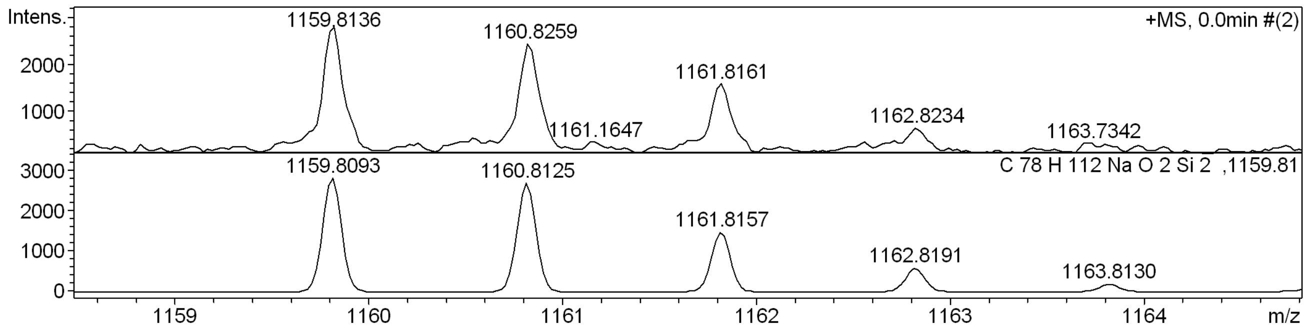

Supplementary Figure 16. Mass spectra (ESI) and data of (R_p)-**6**. Top: experimental spectrum and bottom: theoretical spectrum.

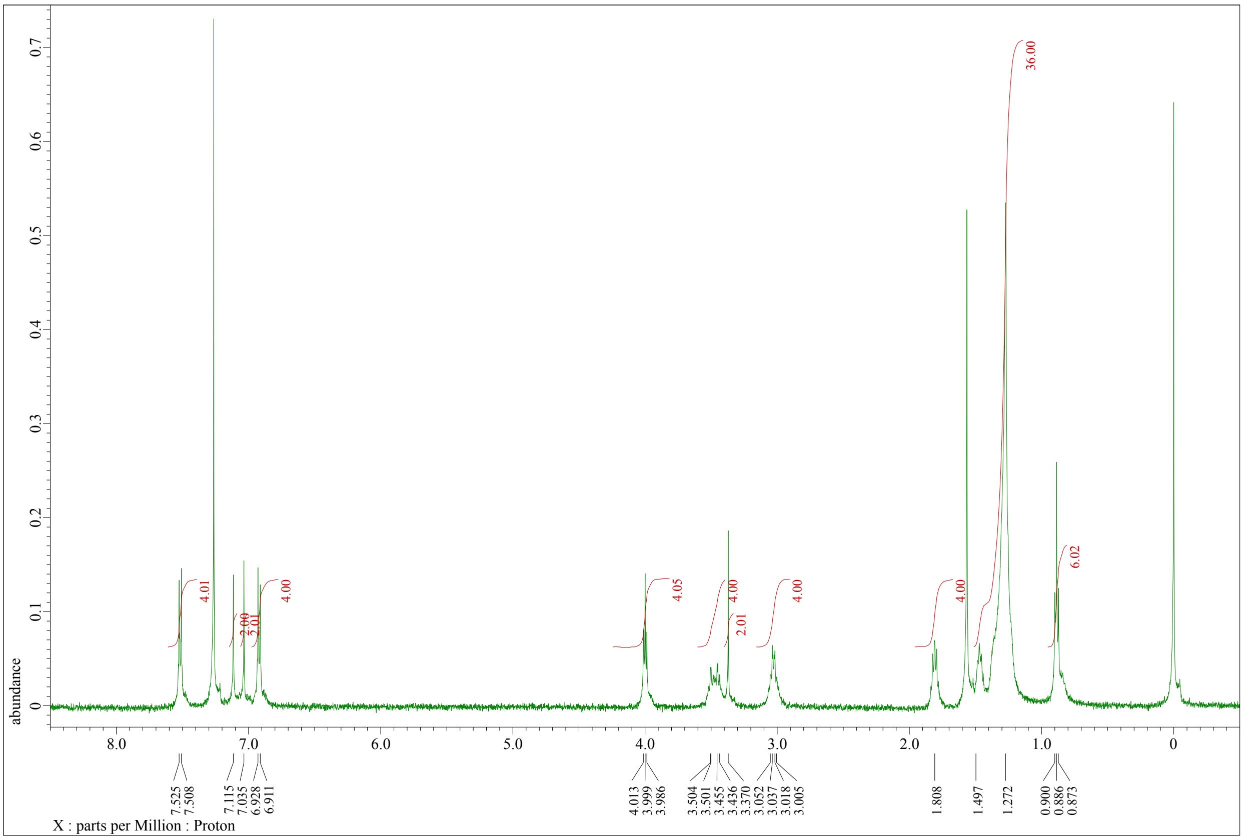

Supplementary Figure 17. ^{1}H NMR spectrum of (S_p)-**7** in $CDCl_3$.

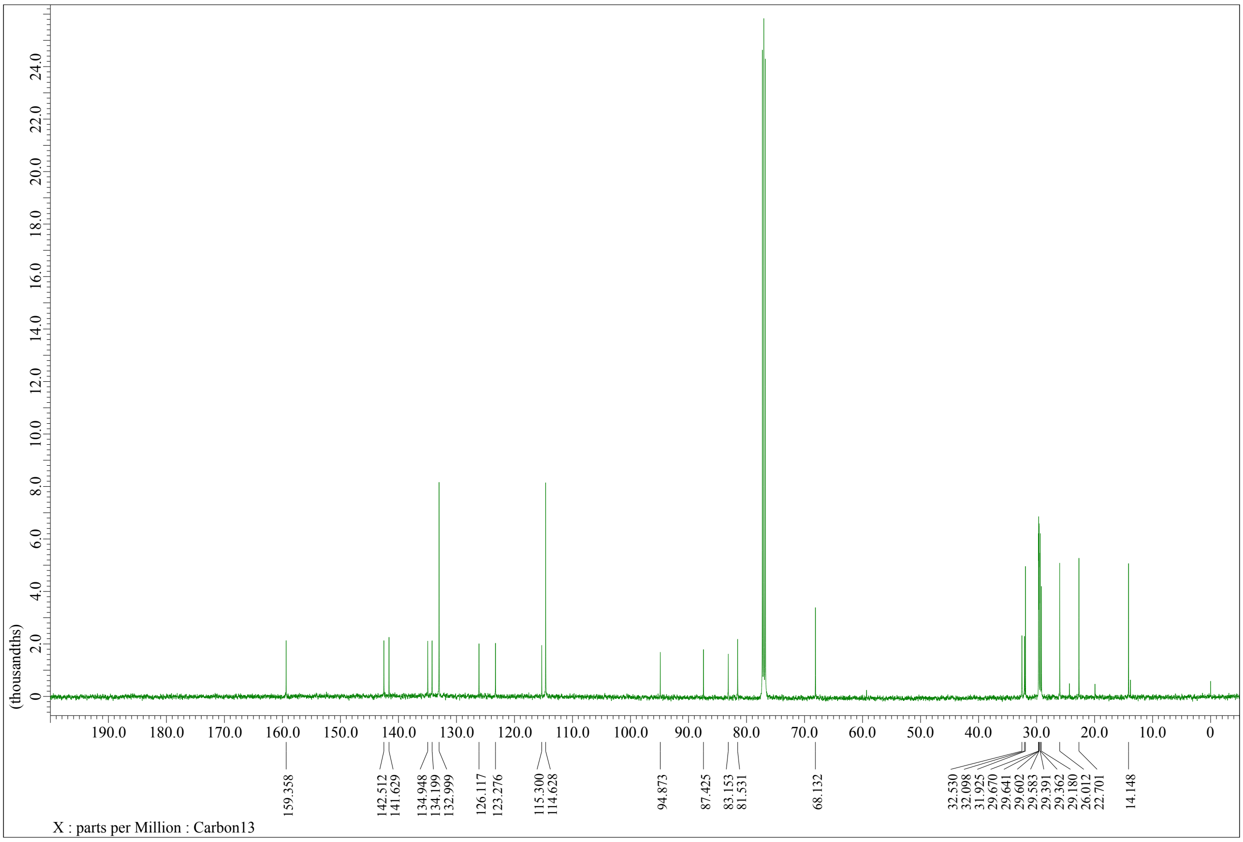

Supplementary Figure 18. ^{13}C NMR spectrum of (S_p)-**7** in $CDCl_3$.

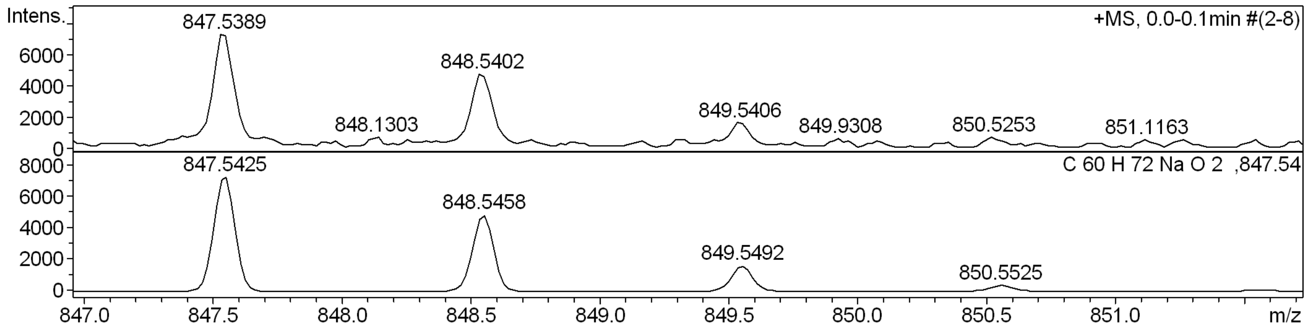

Supplementary Figure 19. Mass spectra (ESI) and data of (S_p)-**7**. Top: experimental spectrum and bottom: theoretical spectrum.

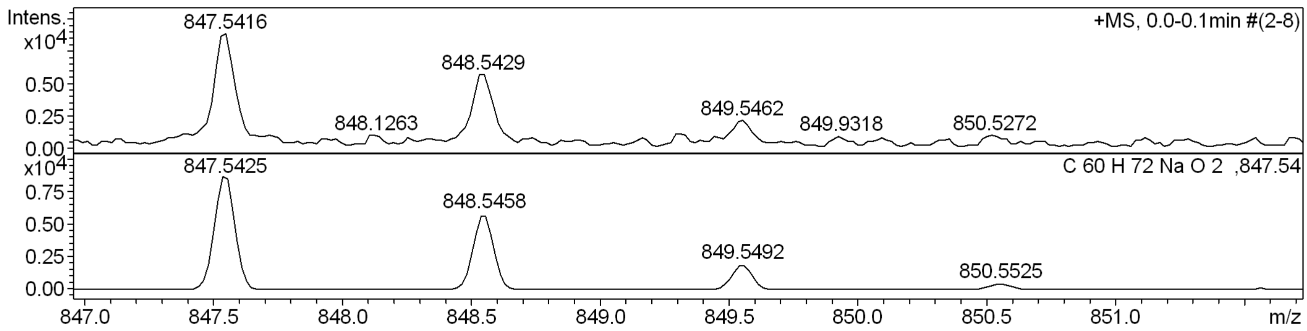

Supplementary Figure 20. Mass spectra (ESI) and data of (R_p)-**7**. Top: experimental spectrum and bottom: theoretical spectrum.

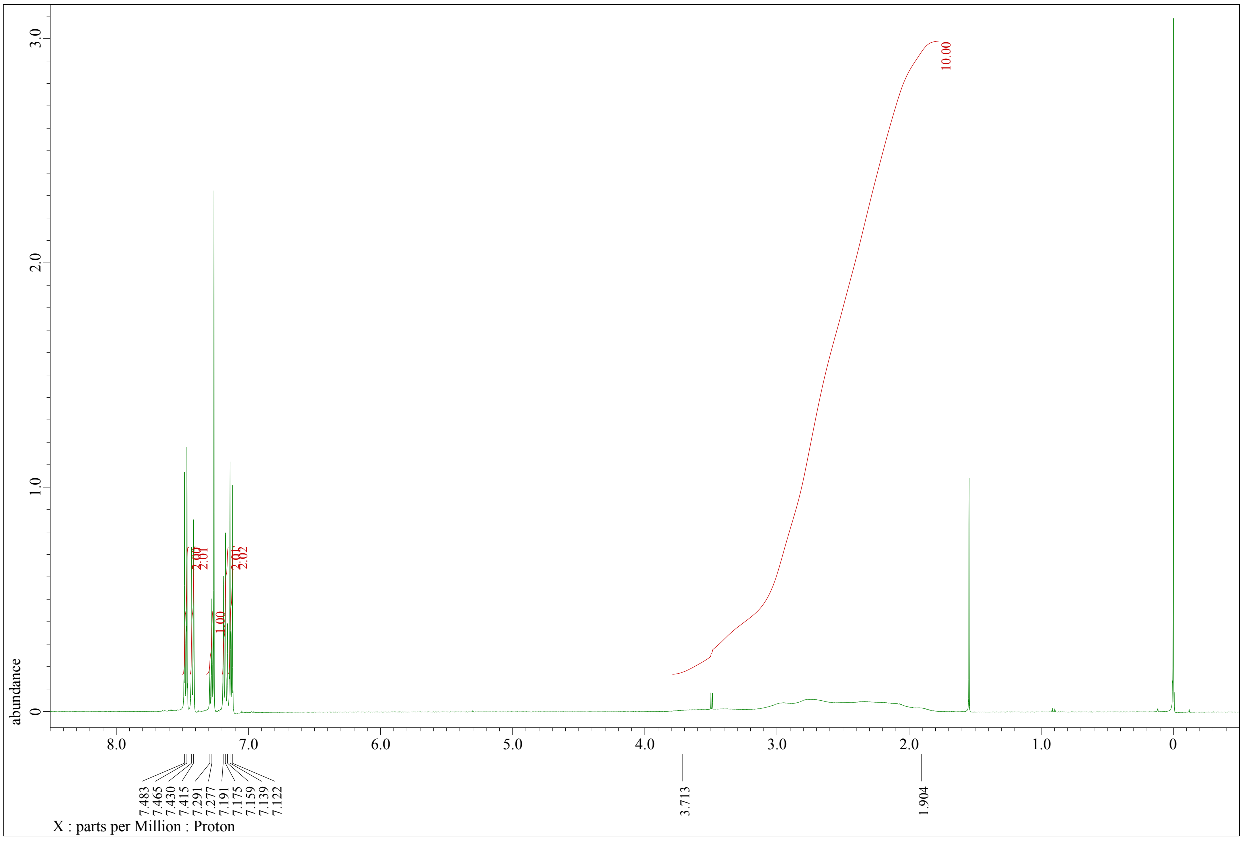

Supplementary Figure 21. ^{1}H NMR spectrum of **10** in $CDCl_3$.

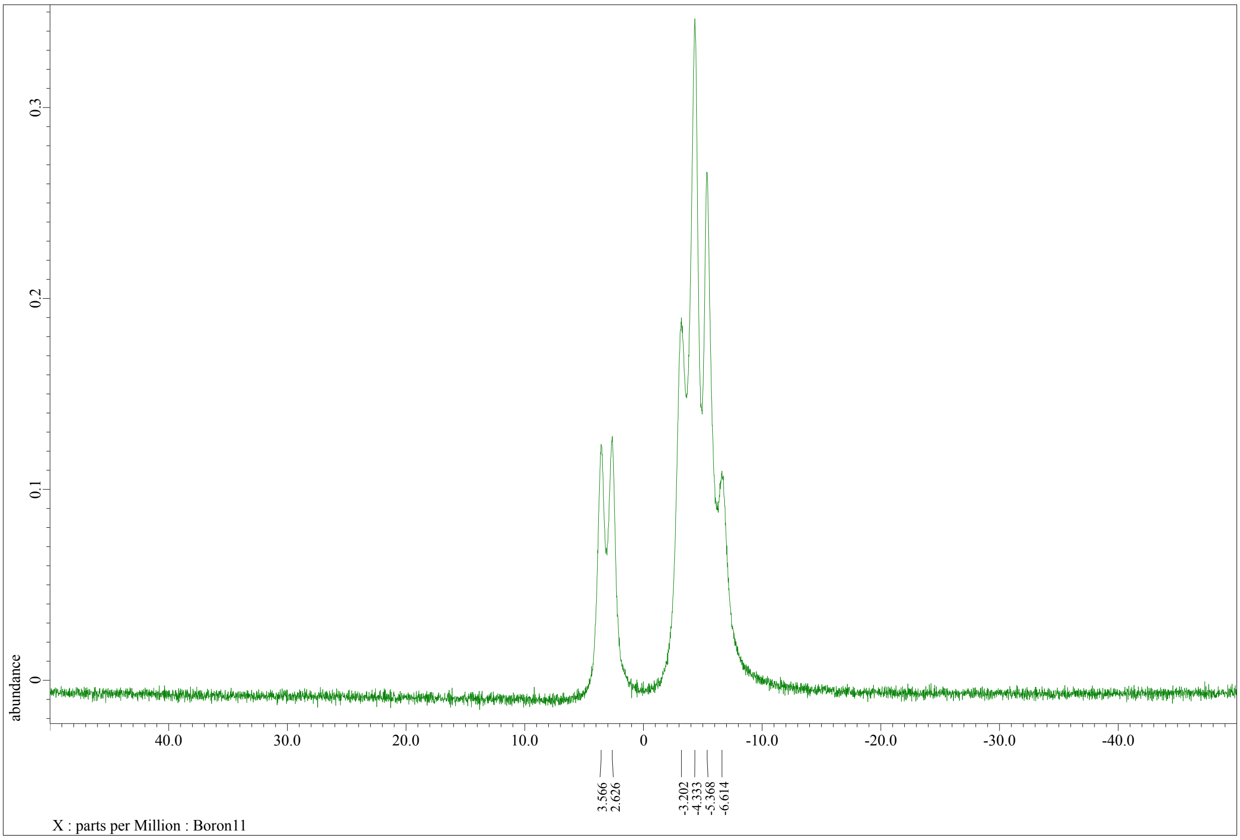

Supplementary Figure 22. ^{11}B NMR spectrum of **10** in $CDCl_3$.

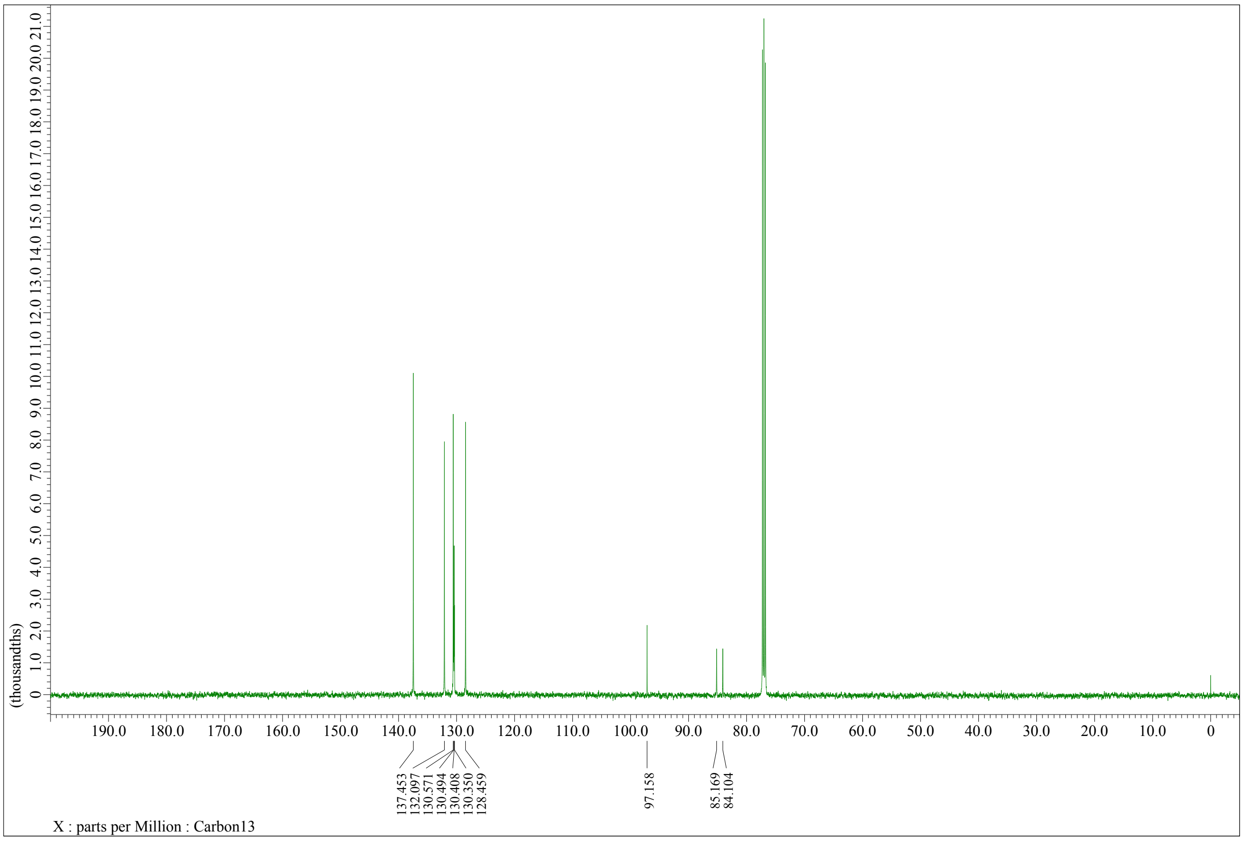

Supplementary Figure 23. ^{13}C NMR spectrum of **10** in $CDCl_3$.

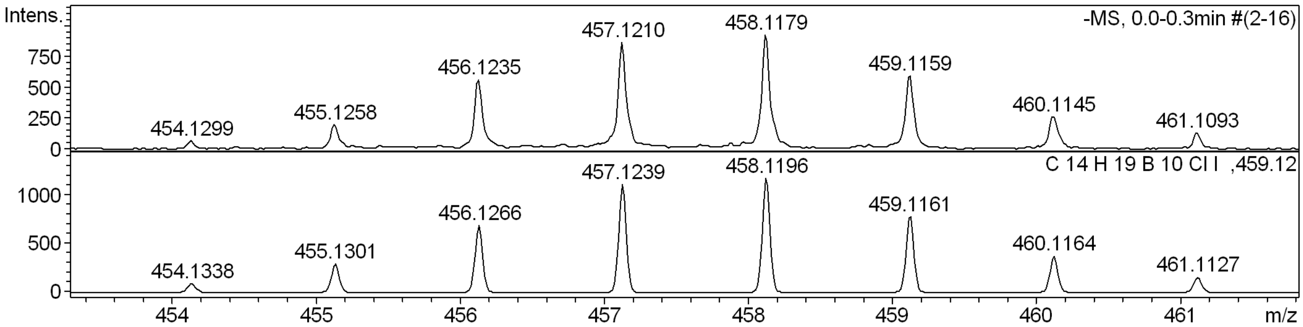

Supplementary Figure 24. Mass spectra (ESI) and data of **10**. Top: experimental spectrum and bottom: theoretical spectrum.

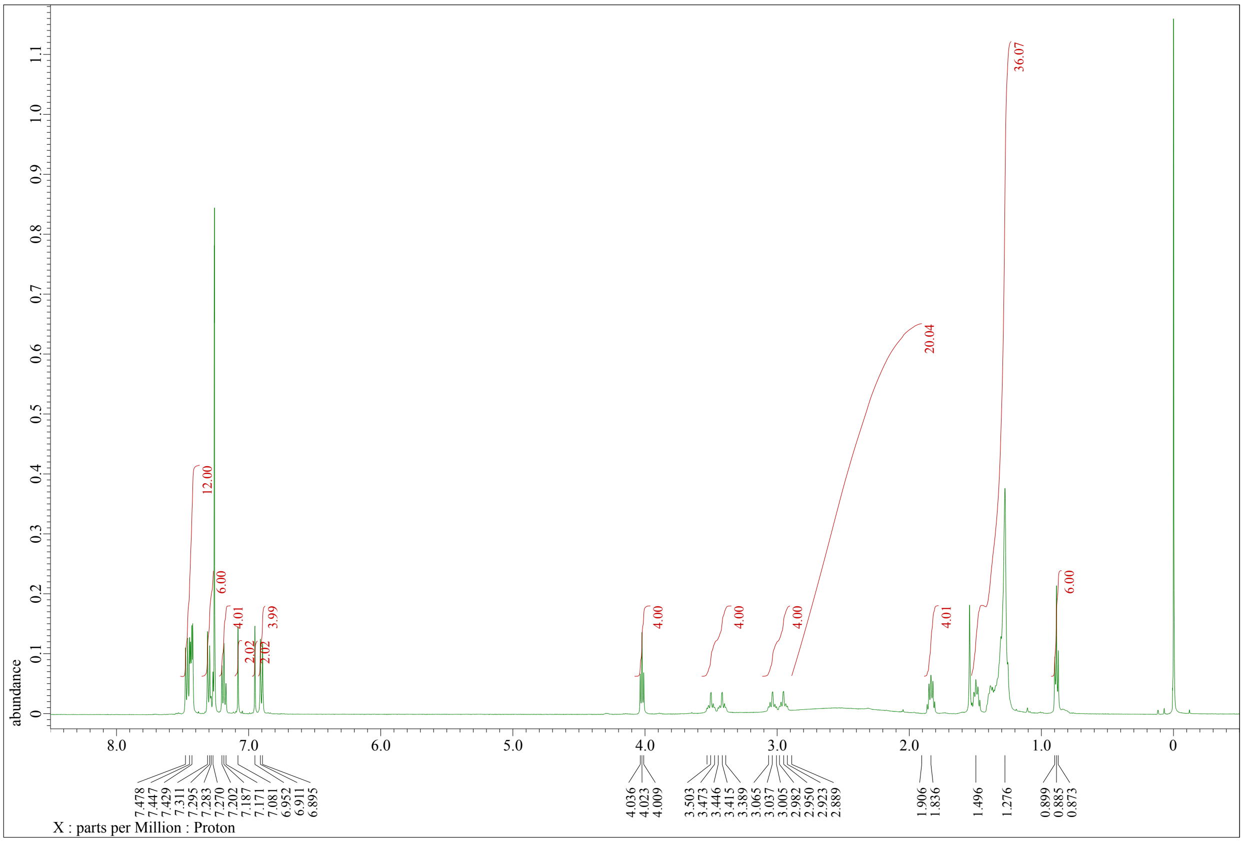

Supplementary Figure 25. ^{1}H NMR spectrum of (S_p)-**11** in $CDCl_3$.

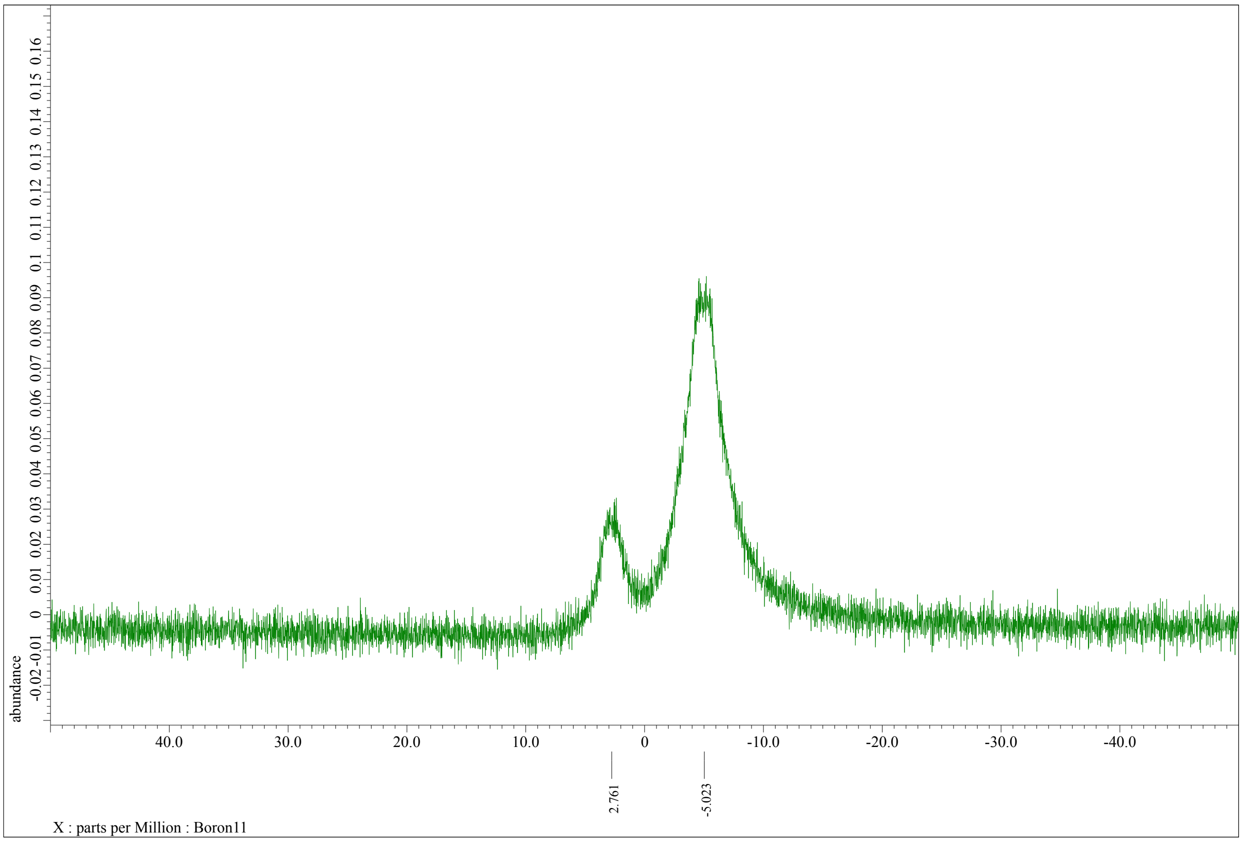

Supplementary Figure 26. ^{11}B NMR spectrum of (S_p)-**11** in $CDCl_3$.

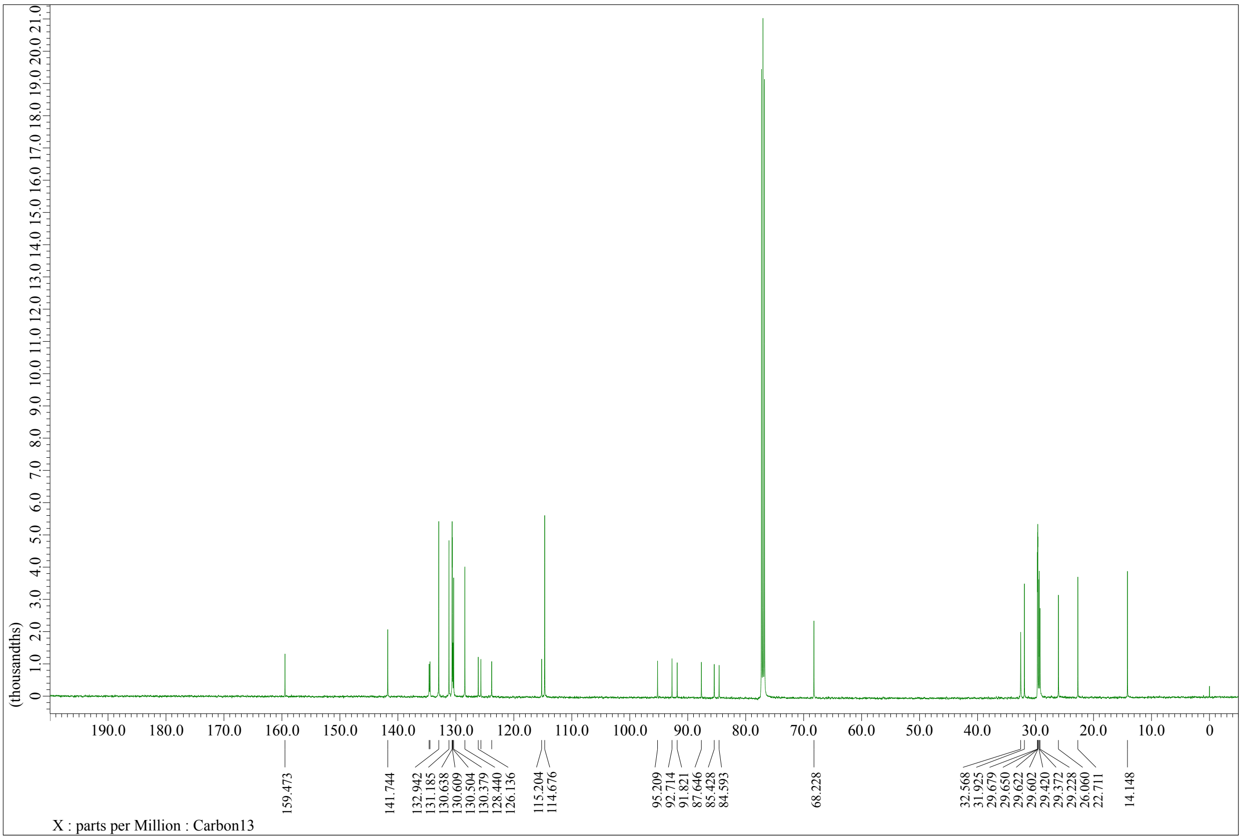

Supplementary Figure 27. ^{13}C NMR spectrum of (S_p)-**11** in $CDCl_3$.

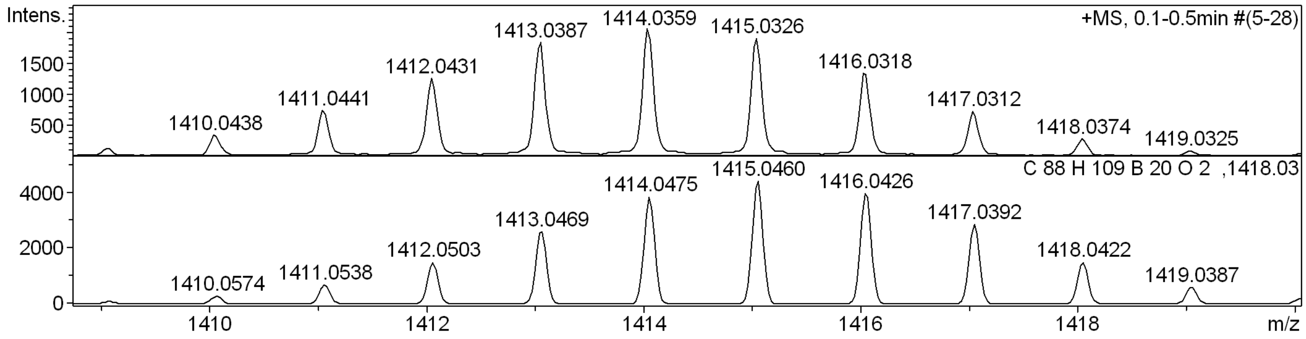

Supplementary Figure 28. Mass spectra (ESI) and data of (S_p)-**11**. Top: experimental spectrum and bottom: theoretical spectrum.

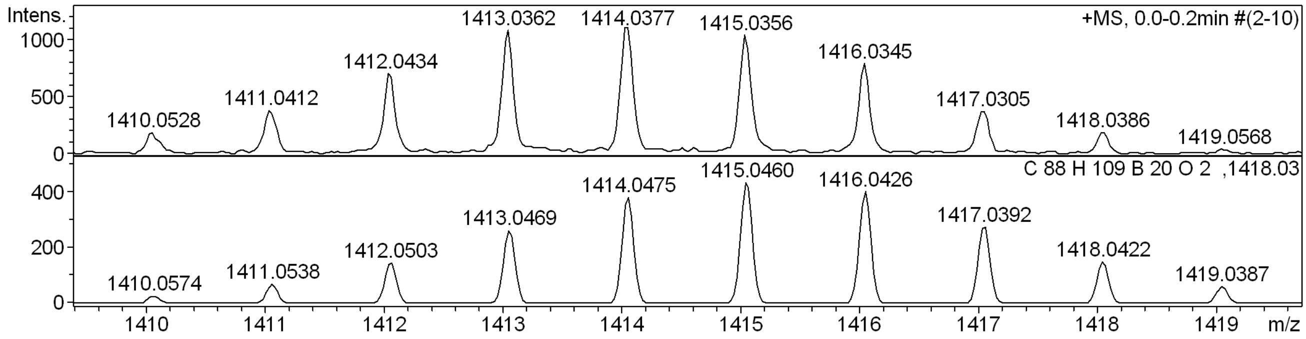

Supplementary Figure 29. Mass spectra (ESI) and data of (R_p)-**11**. Top: experimental spectrum and bottom: theoretical spectrum.

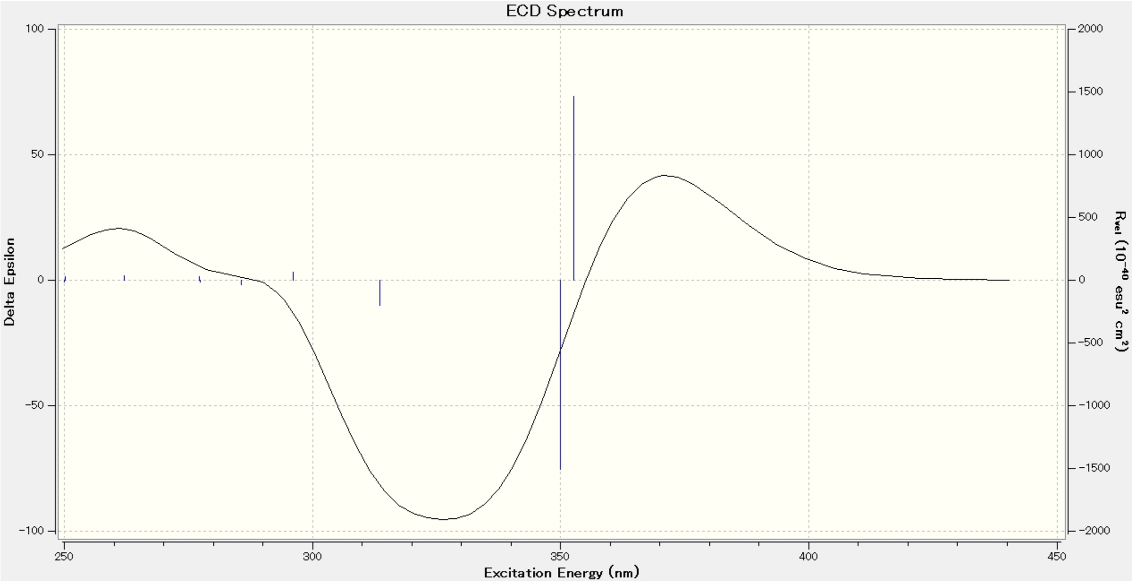

Supplementary Figure 30. Simulated ECD (half-width at half height = 0.20 eV) spectrum of the (S_p)-**11** model by TD-DFT calculation (TD- CAM-B3LYP/6-31G(d)//CAM-B3LYP/6-31G(d)).

Supplementary Table 1. Selected data for excitation energy, major configuration, coefficient, oscillator strength, and rotatory strengths for the (S_p)-**11** model.[a]

State	Excitation energy / eV (/ nm)	Major Configuration	Coefficient	Oscillator strength	Rotatory Strengths / 10^{-40} esu^2 cm^2
S_1	3.52 (352)	H→L	0.63065	2.1597	1467.7564
S_2	3.54 (350)	H–1→L	0.58185	0.7857	-1504.1781
S_3	3.95 (313)	H→L+1	0.51880	0.1554	-201.7373
S_4	4.19 (296)	H–1→L+1	0.60396	0.6211	67.0656
S_5	4.34 (286)	H–3→L	0.55262	0.0078	-37.6603
S_6	4.47 (277)	H–2→L+1	0.43395	0.0424	-14.9616
S_7	4.47 (277)	H–5→L	0.33829	0.0297	28.0243
S_8	4.73 (262)	H–3→L	0.31392	0.0054	34.7477

[a]Estimated by TD-DFT calculations (TD-CAM-B3LYP/6-31G(d)) based on optimized structures determined by DFT calculation (CAM-B3LYP /6-31G(d)). H and L denote HOMO and LUMO.

Supplementary Table 2. Cartesian coordinate of the (S_p)-**11** model in the ground state (CAM-B3LYP/6-31G(d)).

atom	x	y	z
C	1.468744	1.118561	1.290303
C	0.221848	0.469507	1.377549
C	-0.868115	1.238422	1.76275
C	-0.823002	2.639215	1.760006
C	0.357663	3.287485	1.347367
C	1.511795	2.51938	1.272369
H	-1.830709	0.759532	1.910863
H	2.454513	2.998684	1.028707
C	-0.000525	-0.906984	0.795346
H	0.763372	-1.614135	1.130549
H	-0.967055	-1.28115	1.142031
C	0.327205	4.66383	0.724489
H	1.352259	5.03373	0.642784
H	-0.229916	5.372932	1.343187
C	-2.022464	3.376205	1.989475
C	-3.046509	3.991714	2.182511
C	-4.26362	4.700059	2.412432
C	-5.457555	4.00655	2.674363
C	-4.30169	6.096153	2.382204
C	-6.636593	4.687817	2.895116
H	-5.44384	2.922215	2.703774
C	-5.48472	6.790549	2.602382
H	-3.38787	6.646542	2.184591
C	-6.65992	6.08593	2.860485
H	-7.562478	4.16197	3.100436
H	-5.477716	7.872922	2.572155
C	0.000518	-0.907006	-0.795323
C	-0.221865	0.46948	-1.377548
H	-0.76338	-1.614171	-1.130501
H	0.967046	-1.281171	-1.142002
C	-1.468779	1.118507	-1.290339
C	0.868092	1.238398	-1.762734
C	-1.511841	2.519326	-1.272426
C	0.822962	2.639194	-1.760019
H	1.830683	0.759498	-1.910833
C	-0.357725	3.287442	-1.347419
H	-2.454563	2.998645	-1.028805
C	2.022429	3.376179	-1.989508
C	-0.327297	4.663808	-0.724577
C	3.046492	3.991656	-2.182569
H	-1.352353	5.033698	-0.642889
H	0.229825	5.372899	-1.343291
C	4.263686	4.699869	-2.412498
C	5.457561	4.006211	-2.674303
C	4.301887	6.095961	-2.382402
C	6.636676	4.687339	-2.895067
H	5.44374	2.921873	-2.703611
C	5.484997	6.790216	-2.602595
H	3.388114	6.646463	-2.184887
H	7.562519	4.161378	-3.100293
H	5.478095	7.872592	-2.572478
C	6.660144	6.085455	-2.860572
O	7.866688	6.65946	-3.089365
C	7.951631	8.068714	-3.074818
H	8.994315	8.310083	-3.281691
H	7.316337	8.517337	-3.847702
H	7.673004	8.478036	-2.096464
O	-7.866404	6.660078	3.089278
C	-7.951207	8.069335	3.074593
H	-8.993855	8.310833	3.281497
H	-7.315828	8.517979	3.847397
H	-7.672591	8.478535	2.096183
C	-2.662519	0.381943	-1.031421
C	-3.680781	-0.233259	-0.810897
C	-4.890789	-0.943052	-0.553037
C	-6.088263	-0.253855	-0.324958
C	-4.910774	-2.34181	-0.514365
C	-7.262868	-0.943882	-0.079214
H	-6.088663	0.830445	-0.340131
C	-6.088452	-3.026617	-0.263916
H	-3.991266	-2.891798	-0.681503
H	-8.171383	-0.384062	0.10461
C	-7.286171	-2.341032	-0.052269
H	-6.065795	-4.107574	-0.228975
C	2.662483	0.382015	1.031349
C	3.680752	-0.233161	0.810796
C	4.890771	-0.94293	0.552969
C	6.088273	-0.253746	0.325015
C	4.910737	-2.341691	0.514194
C	7.26288	-0.943781	0.079302
H	6.088691	0.830553	0.340258
C	6.088412	-3.026505	0.263773
H	3.991201	-2.891662	0.681233
H	8.171418	-0.38397	-0.10443
C	7.286158	-2.340929	0.052261
H	6.065743	-4.107462	0.228743
C	-8.570444	-3.067227	0.247638
C	-9.467728	-3.745695	-1.037355
B	-8.641627	-4.766654	0.066931
B	-10.03318	-2.329362	-0.261026
B	-8.76086	-4.011559	1.655496

B	-9.629675	-2.491043	1.449137
C	-8.92728	-3.659506	-2.441559
B	-10.2674	-5.149357	-0.501799
B	-11.1369	-3.629122	-0.707261
H	-7.687827	-5.344089	-0.311129
B	-9.849441	-5.330379	1.206776
H	-9.971935	-1.299776	-0.828732
B	-11.26272	-2.85085	0.875387
H	-7.824782	-4.082574	2.376602
B	-10.47929	-3.910729	2.068531
H	-9.294775	-1.520215	2.037366
C	-9.075572	-2.50448	-3.210927
H	-10.39229	-6.026858	-1.285806
B	-11.40795	-4.615674	0.737459
H	-11.85031	-3.458693	-1.636254
H	-9.740108	-6.405649	1.695883
H	-12.17012	-2.13089	1.131663
H	-10.82932	-3.956121	3.201311
H	-9.575577	-1.637199	-2.800823
C	-8.593962	-2.454948	-4.512762
C	-7.823691	-4.719007	-4.317624
H	-12.43752	-5.184422	0.893212
H	-8.717956	-1.545934	-5.092379
C	-7.963325	-3.559598	-5.070375
H	-7.342848	-5.593538	-4.74391
H	-7.588315	-3.519491	-6.088085
C	8.57042	-3.067144	-0.24762
C	9.467626	-3.745736	1.037452
B	8.641516	-4.766558	-0.066926
B	10.03315	-2.329416	0.261183
B	8.760875	-4.011441	-1.655475
B	9.629754	-2.490989	-1.449021
C	8.927147	-3.659523	2.441637
B	10.267227	-5.149421	0.501877
B	11.13681	-3.629237	0.707432
H	7.68766	-5.343977	0.311015
B	9.849348	-5.330343	-1.206722
H	9.971932	-1.299844	0.828918
B	11.26275	-2.850914	-0.875177
H	7.824817	-4.082413	-2.376612
B	10.479325	-3.910707	-2.068406
H	9.294963	-1.520097	-2.037205
C	9.076015	-2.504694	3.211194
H	10.391964	-6.02697	1.285854
B	11.407878	-4.615749	-0.737307
H	11.850165	-3.45884	1.636474
H	9.739973	-6.405593	-1.695865
H	12.170201	-2.130992	-1.131368
H	10.829422	-3.956092	-3.201165
H	9.576486	-1.637613	2.801235
C	8.594404	-2.455122	4.513026
C	7.823002	-4.718765	4.317519
H	12.437418	-5.184549	-0.893039
H	8.718853	-1.546265	5.092793
C	7.963193	-3.55954	5.070449
H	7.341722	-5.593123	4.743665
H	7.588179	-3.519404	6.088157
C	8.301225	-4.768864	3.015277
H	8.195723	-5.68454	2.446798
C	-8.301906	-4.769064	-3.015378
H	-8.196825	-5.684871	-2.44703

Supplementary Table 3. Cartesian coordinate of the (S_p)-**11** model in the S_1 state (TD-CAM-B3LYP/6-31G(d)).

atom	x	y	z
C	1.473738	1.172324	1.095247
C	0.180575	0.536959	1.211829
C	-0.872477	1.310261	1.609902
C	-0.79168	2.74023	1.644009
C	0.413745	3.376468	1.16761
C	1.532504	2.603215	1.039068
H	-1.850755	0.860792	1.746954
H	2.465185	3.053095	0.715115
C	-0.044483	-0.836227	0.633906
H	0.701845	-1.552074	0.99222
H	-1.025936	-1.193679	0.954719
C	0.361098	4.752854	0.556321
H	1.383303	5.113475	0.419834
H	-0.15729	5.463966	1.207121
C	-1.89988	3.490353	2.009906
C	-2.885636	4.145722	2.335175
C	-4.014584	4.883639	2.715809
C	-5.190106	4.23424	3.166391
C	-4.016673	6.29219	2.66214
C	-6.296686	4.959185	3.538545
H	-5.205954	3.150931	3.215928
C	-5.129061	7.024174	3.036549
H	-3.124491	6.806281	2.320879
C	-6.279436	6.36114	3.477846
H	-7.200945	4.471771	3.885878
H	-5.096056	8.105242	2.983724
C	0.006143	-0.830874	-0.952456
C	-0.234323	0.550289	-1.511853
H	-0.733895	-1.550702	-1.313548
H	0.989767	-1.179391	-1.277152
C	-1.48668	1.180853	-1.375965
C	0.843135	1.335174	-1.897513
C	-1.541453	2.585632	-1.330249
C	0.777149	2.740785	-1.901928
H	1.807328	0.86785	-2.069217
C	-0.40393	3.369369	-1.461545
H	-2.481049	3.053251	-1.053768
C	1.951918	3.496835	-2.179666
C	-0.372535	4.749363	-0.850488
C	2.954127	4.131351	-2.424654
H	-1.398942	5.100454	-0.714708
H	0.138633	5.467802	-1.497387
C	4.142476	4.864192	-2.715791
C	5.340067	4.195974	-3.024432
C	4.149624	6.261436	-2.701636
C	6.491502	4.90191	-3.30499
H	5.351375	3.111422	-3.041722
C	5.304919	6.980379	-2.982132
H	3.232965	6.793087	-2.468776
H	7.419406	4.394905	-3.545591
H	5.273667	8.062617	-2.962712
C	6.483671	6.300405	-3.285765
O	7.665264	6.899922	-3.574629
C	7.717212	8.310489	-3.57627
H	8.74359	8.574355	-3.831739
H	7.037579	8.736866	-4.323733
H	7.472753	8.723011	-2.590072
O	-7.416752	6.976778	3.864558
C	-7.468841	8.389678	3.8321
H	-8.464613	8.661084	4.181855
H	-6.717469	8.832013	4.496222
H	-7.32476	8.771046	2.814677
C	-2.663516	0.426289	-1.10742
C	-3.672731	-0.2069	-0.888552
C	-4.866834	-0.940556	-0.633043
C	-6.086937	-0.278661	-0.44215
C	-4.852445	-2.338919	-0.558258
C	-7.246669	-0.992749	-0.196785
H	-6.116168	0.804391	-0.486857
C	-6.015737	-3.047588	-0.30876
H	-3.916512	-2.86898	-0.696251
H	-8.172242	-0.452363	-0.041965
C	-7.234725	-2.389104	-0.133266
H	-5.965386	-4.126394	-0.245263
C	2.621689	0.420938	0.902405
C	3.643689	-0.238337	0.727008
C	4.814061	-0.979984	0.531131
C	6.064481	-0.339377	0.370463
C	4.786245	-2.392179	0.47567
C	7.213921	-1.07505	0.179152
H	6.109249	0.743865	0.395283
C	5.943846	-3.115744	0.28299
H	3.837387	-2.906137	0.584392
H	8.153027	-0.552613	0.044592
C	7.185068	-2.47803	0.140802
H	5.88172	-4.194998	0.233483
C	-8.503408	-3.14061	0.167349
C	-9.369422	-3.870095	-1.113211
B	-8.530374	-4.844731	0.02232
B	-9.978454	-2.452261	-0.374427
B	-8.687387	-4.05941	1.592803

B	-9.591421	-2.566199	1.343436
C	-8.814937	-3.800851	-2.512773
B	-10.13952	-5.281367	-0.555646
B	-11.04447	-3.78871	-0.804046
H	-7.558003	-5.405846	-0.332296
B	-9.736959	-5.414716	1.160963
H	-9.937581	-1.433998	-0.963944
B	-11.20796	-2.979557	0.759551
H	-7.758618	-4.091395	2.326078
B	-10.41248	-3.993255	1.984252
H	-9.288366	-1.574483	1.913991
C	-8.983613	-2.667202	-3.309253
H	-10.23317	-6.17877	-1.32127
B	-11.30749	-4.750141	0.658835
H	-11.75135	-3.656331	-1.7442
H	-9.606424	-6.476031	1.674762
H	-12.13612	-2.277371	0.989807
H	-10.7746	-4.022951	3.113823
H	-9.510262	-1.804286	-2.924032
C	-8.4882	-2.633827	-4.606406
C	-7.662588	-4.87237	-4.352367
H	-12.32426	-5.341244	0.815647
H	-8.628464	-1.741228	-5.207444
C	-7.822993	-3.733769	-5.132239
H	-7.154396	-5.743265	-4.753629
H	-7.437075	-3.706298	-6.146285
C	8.441676	-3.253744	-0.104676
C	9.304069	-3.933329	1.233556
B	8.451331	-4.946266	0.145255
B	9.924192	-2.56555	0.417923
B	8.628243	-4.257202	-1.469346
B	9.550683	-2.763727	-1.296702
C	8.741875	-3.778793	2.621786
B	10.052331	-5.379686	0.754067
B	10.976495	-3.888408	0.925482
H	7.469771	-5.475169	0.524552
B	9.654586	-5.599791	-0.95456
H	9.901258	-1.516092	0.951274
B	11.156532	-3.166797	-0.678452
H	7.704861	-4.321961	-2.207456
B	10.356329	-4.236308	-1.851228
H	9.268609	-1.799149	-1.922188
C	8.922406	-2.605407	3.355784
H	10.127866	-6.238436	1.565072
B	11.233253	-4.931263	-0.481717
H	11.682861	-3.710602	1.858778
H	9.511773	-6.686183	-1.40985
H	12.095793	-2.490662	-0.940263
H	10.724901	-4.333711	-2.975077
H	9.464596	-1.773371	2.926728
C	8.419674	-2.492908	4.645679
C	7.561167	-4.729376	4.509507
H	12.242745	-5.543622	-0.600782
H	8.569828	-1.57055	5.197466
C	7.73453	-3.552048	5.226755
H	7.036802	-5.568935	4.954504
H	7.342897	-3.462895	6.235116
C	8.060459	-4.842062	3.219233
H	7.92756	-5.770785	2.678353
C	-8.154651	-4.906172	-3.054774
H	-8.032681	-5.806206	-2.465019

Supplementary Table 4. Cartesian coordinate of the corresponding X-shaped molecule in the ground state (CAM-B3LYP/6-31G(d)).

atom	x	y	z
C	-1.107272	-1.259591	-1.606492
C	-1.147756	0.141292	-1.602869
C	-0.007902	0.906339	-1.394648
C	0.121854	-1.911758	-1.389324
H	-2.119996	0.623483	-1.595256
C	0.007991	0.90639	1.394649
C	1.147792	0.141275	1.602896
C	1.107223	-1.259608	1.606549
C	-0.121941	-1.911708	1.389421
H	2.120065	0.623401	1.595262
C	-0.191757	-3.289031	0.772055
H	0.473205	-3.991776	1.281883
H	-1.211544	-3.667942	0.876197
C	0.191587	-3.289066	-0.771917
H	1.211353	-3.668037	-0.876047
H	-0.473409	-3.991796	-1.281722
C	0.129631	2.283476	0.784665
H	-0.572708	2.987692	1.239529
H	1.138682	2.66022	0.969714
C	-0.129449	2.283457	-0.784713
H	-1.138475	2.660258	-0.969778
H	0.572931	2.987616	-1.239604
C	1.275483	-1.146556	-1.496978
C	1.235718	0.254337	-1.503542
C	-1.23567	0.254466	1.503534
C	-1.27552	-1.146426	1.497036
H	-2.24341	-1.628588	1.404572
C	-2.45875	0.984816	1.424015
C	2.458842	0.984612	-1.424112
C	3.504914	1.588369	-1.348128
C	-3.504793	1.58861	1.347952
C	-2.332865	-1.989858	-1.635096
C	-3.381191	-2.593635	-1.65263
H	2.243342	-1.628779	-1.404512
C	-4.750615	2.278178	1.2538
C	-7.16036	2.227747	1.017102
C	-4.809081	3.67309	1.296614
C	-7.203229	3.624848	1.06044
H	-8.093945	1.686746	0.908668
H	-3.889595	4.237915	1.40849
C	4.750687	2.278014	-1.253913
C	7.160425	2.22773	-1.017108
C	4.809073	3.67293	-1.29673
C	7.203216	3.624834	-1.06045
H	8.094033	1.68678	-0.908631
H	3.88956	4.237702	-1.40865
C	-4.629579	-3.287644	-1.671088
C	-7.04813	-3.246975	-1.692789
C	-4.668912	-4.68813	-1.679908
H	-7.975826	-2.683139	-1.699011
H	-3.736915	-5.243377	-1.677421
O	8.437088	4.179851	-0.958635
O	-8.437145	4.179793	0.958668
C	8.542148	5.586773	-0.998045
H	9.604247	5.813094	-0.90202
H	7.996737	6.054224	-0.169473
H	8.170823	5.99142	-1.947171
C	-8.542289	5.586701	0.998098
H	-9.604407	5.812963	0.90213
H	-7.996954	6.054204	0.169503
H	-8.17094	5.991369	1.947206
C	-5.885419	-5.354882	-1.693798
H	-5.903118	-6.440311	-1.700646
C	-5.835537	-2.57343	-1.677662
H	-5.806752	-1.488885	-1.671474
C	-5.953616	1.565645	1.111904
H	-5.924756	0.481659	1.077466
C	-6.019876	4.348208	1.200868
H	-6.027692	5.430398	1.238135
C	5.953722	1.565554	-1.11196
H	5.924932	0.481567	-1.077512
C	6.019823	4.348121	-1.200935
H	6.027573	5.430312	-1.238211
C	2.332787	-1.989935	1.635133
C	3.3811	-2.593734	1.652636
C	4.629537	-3.287657	1.6711
C	5.835444	-2.573358	1.677765
C	4.668968	-4.68814	1.679838
C	7.048083	-3.246817	1.692903
H	5.806582	-1.488814	1.671642
C	5.885522	-5.354806	1.693738
H	3.737012	-5.243453	1.677281
H	7.975738	-2.682915	1.699197
H	5.903297	-6.440235	1.700521
C	-7.077771	-4.638073	-1.700437
H	-8.02817	-5.162413	-1.71221
C	7.077824	-4.637913	1.700468
H	8.028259	-5.162186	1.712251

Supplementary Table 5. Cartesian coordinate of the corresponding X-shaped molecule in the S_1 state (TD-CAM-B3LYP/6-31G(d)).

atom	x	y	z
C	-1.077216	-1.339751	-1.84559
C	-1.087887	0.092741	-1.836608
C	0.051073	0.851457	-1.650387
C	0.18172	-1.978416	-1.570602
H	-2.057998	0.584503	-1.833423
C	0.020151	0.93425	1.125965
C	1.118112	0.141712	1.381989
C	1.014565	-1.279823	1.449985
C	-0.243622	-1.919101	1.171948
H	2.111641	0.576673	1.374951
C	-0.311012	-3.312194	0.59268
H	0.284921	-4.02107	1.178164
H	-1.350392	-3.649675	0.620208
C	0.206068	-3.345053	-0.920288
H	1.230123	-3.732226	-0.927642
H	-0.424087	-4.065118	-1.453638
C	0.184463	2.296579	0.495825
H	-0.499874	3.030052	0.936913
H	1.205028	2.642625	0.679859
C	-0.071037	2.24953	-1.082262
H	-1.077214	2.633659	-1.279947
H	0.643431	2.942698	-1.539443
C	1.338217	-1.224813	-1.62232
C	1.332863	0.204804	-1.701977
C	-1.269373	0.305038	1.246043
C	-1.365275	-1.121052	1.22724
H	-2.348684	-1.56076	1.100666
C	-2.437538	1.064453	1.290043
C	2.532528	0.928467	-1.624422
C	3.58443	1.558537	-1.544348
C	-3.477788	1.715303	1.359125
C	-2.275146	-2.061345	-1.912703
C	-3.33211	-2.690256	-1.956989
H	2.29526	-1.712344	-1.45103
C	-4.671814	2.446803	1.433618
C	-7.096711	2.5048	1.616962
C	-4.660271	3.8619	1.397628
C	-7.067317	3.915123	1.580187
H	-8.059484	2.011744	1.699448
H	-3.709888	4.378494	1.311362
C	4.805561	2.266649	-1.472047
C	7.248284	2.274468	-1.377064
C	4.844257	3.677768	-1.416026
C	7.262029	3.677332	-1.319904
H	8.197933	1.747523	-1.36529
H	3.909468	4.230403	-1.43683
C	-4.547165	-3.401826	-2.027493
C	-6.974169	-3.435789	-2.311153
C	-4.585679	-4.813201	-1.879254
H	-7.902837	-2.89632	-2.484392
H	-3.654124	-5.348053	-1.717197
O	8.501783	4.259899	-1.246241
O	-8.269252	4.522103	1.656862
C	8.568847	5.672706	-1.206993
H	9.630604	5.923868	-1.158623
H	8.0612	6.080273	-0.321444
H	8.131309	6.126071	-2.107327
C	-8.333446	5.94653	1.617334
H	-9.392755	6.194266	1.689286
H	-7.929054	6.334939	0.675325
H	-7.794675	6.390808	2.462537
C	-5.788524	-5.505252	-1.945177
H	-5.787957	-6.586921	-1.830257
C	-5.778926	-2.730564	-2.246998
H	-5.770499	-1.651452	-2.371055
C	-5.924439	1.783757	1.545104
H	-5.94662	0.699361	1.568255
C	-5.837197	4.590324	1.469663
H	-5.797407	5.672522	1.438826
C	6.050323	1.584423	-1.451217
H	6.054089	0.499572	-1.500117
C	6.048521	4.375378	-1.340884
H	6.027836	5.459098	-1.302156
C	2.167039	-2.037465	1.691102
C	3.179374	-2.681212	1.93856
C	4.354	-3.414631	2.220325
C	5.574182	-2.741516	2.460235
C	4.325346	-4.827436	2.265448
C	6.727209	-3.465979	2.73631
H	5.596057	-1.657259	2.4209
C	5.485228	-5.540066	2.543508
H	3.388774	-5.343612	2.080186
H	7.661536	-2.942662	2.917123
H	5.456297	-6.625315	2.576048
C	-6.994573	-4.827528	-2.159925
H	-7.93206	-5.374034	-2.212024
C	6.686927	-4.863712	2.77941
H	7.591346	-5.425435	2.995189
