# Supplementary material for: Aggregation-induced emission from optically active X-shaped molecules based on planar chiral [2.2]paracyclophane
Source: Sci Rep. 2023 Dec 19;13:22647. doi: 10.1038/s41598-023-49120-2 (PMC10730888; doi:10.1038/s41598-023-49120-2)
Supplement: Supplementary file 2 — Supplementary Information 2. [file 41598_2023_49120_MOESM2_ESM.pdf]

Studies on solvatochromism : only for peer-review

(S_p)-**11** was not sufficiently solved in hexane, cyclohexane, toluene, and dioxane; thus, (S_p)-**11** was aggregated in their solutions and exhibited solvatochromism (Figure 1). The data of the aggregates in hexane, cyclohexane, toluene, and dioxane (DIO) are shown in Table 1.

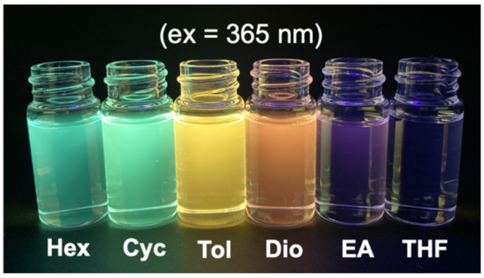

Φ_{PL} 14% 18% 17% 5% 1% 0%

Figure 1. PL of (S_p)-**11** in various solvents such as hexane (Hex), cyclohexane (Cyc), toluene (Tol), dioxane (DIO), ethylacetate (EA), and THF (1.0×10^{-5}). Excitation wavelength = 365 nm.

Table 1. Optical date of (S_P)-**T1** in hexane, cyclohexane, toluene, 1,4-dioxane (1.0×10^{-5}).

solvent	$\lambda_{abs, max}$ / nm	$\varepsilon \times 10^{-5}$ / M^{-1} cm^{-1}	$\lambda_{PL, max}$ / nm[a]	Φ_{PL} / %[b]	τ / ns[c]	Stokes shift / cm	$E_T(30)$[d] / kcal mol^{-1}
hexane	367	0.39	533	14	0.40<br>2.54	8487	31.0
cyclohexane	368	0.38	541	18	0.43<br>3.59	8687	30.9
toluene	366	0.36	591	17	0.73<br>3.72	10402	33.9
1,4-dioxane	368	0.38	623	5	0.43<br>1.44	11122	36.0

[a] Excited at each $\lambda_{abs, max}$.
[b] Absolute PL quantum yield.
[c] PL lifetime monitored around 365 nm; the PL decay was fitted by a double exponential function.
[d] Parameters of solvent polarity.

The UV-vis and PL spectra are shown in Figures 2A and 2B. Main PL peaks were shifted depending on the solvent polarity. The Lippert-Mataga plots suggested that the emission was charge-transfer emission.

(A) UV-vis absorption spectra

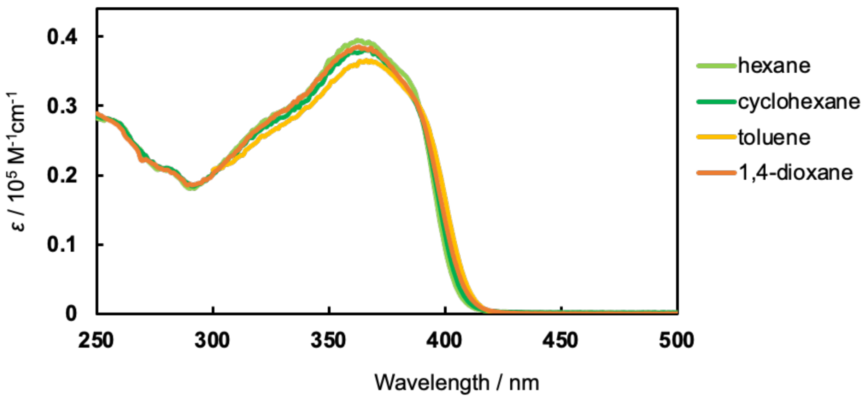

(B) PL spectrum

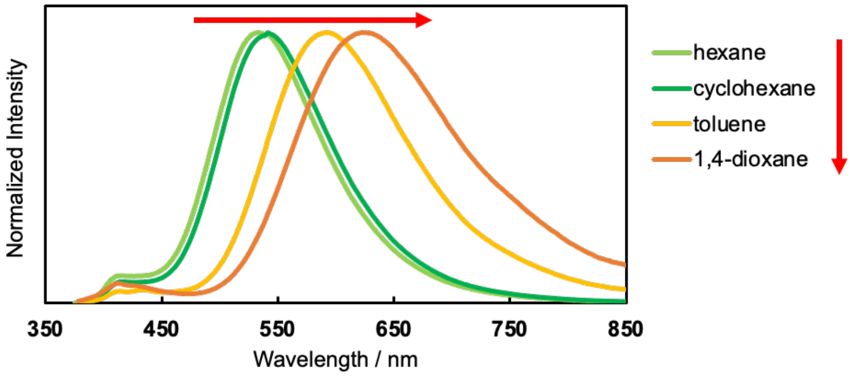

(C)

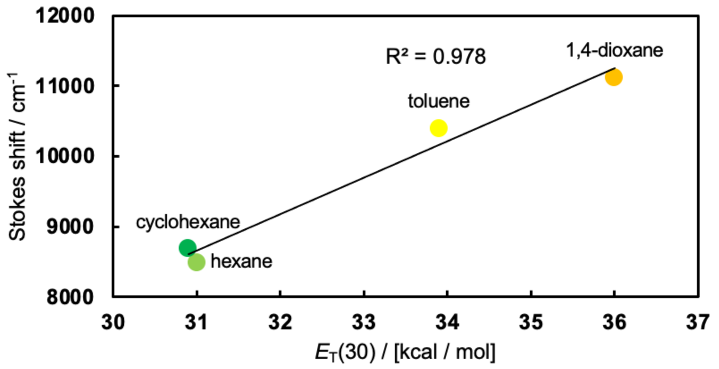

Figure 2. (A) UV-vis absorption spectra of (S_p)-**T1** in various solvents (1.0×10^{-5}). (B) PL spectra of (S_p)-**T1** in various solvents (1.0×10^{-5}). (C) Correlation between the Stokes shift value of (S_p)-**T1** and the solvent polarity parameter $E_T(30)$.

Their chiroptical properties, CD and CPL behaviors, were also investigated, as shown in Figures 2-5.

(A) CD and UV-vis absorption spectra in hexane (B) CPL and PL spectra in hexane

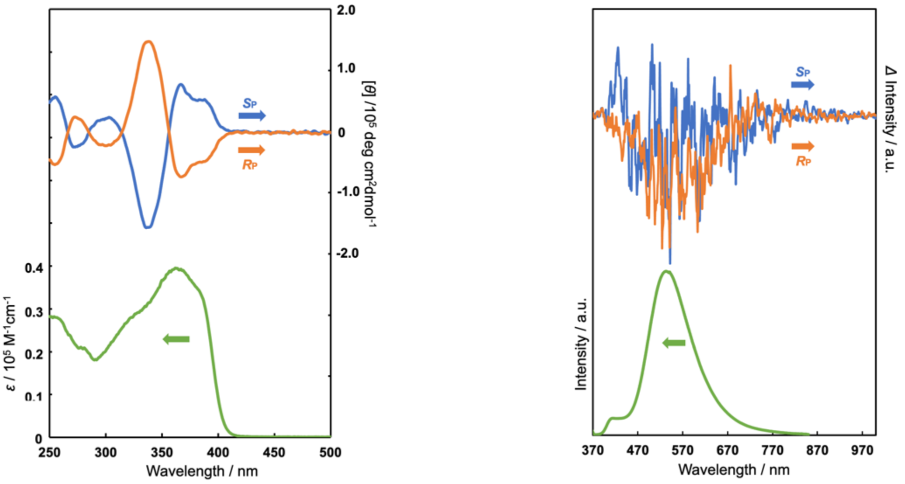

$\lambda_{PL,max}$ = 533 nm, $|g_{lum}|$ ··· not calculated due to the noisy spectra

Figure 2. (A) CD and UV-vis absorption spectra of (R_p)- and (S_p)-**11** in hexane (1.0 × 10^{-5}). (B) CPL and PL spectra of (R_p)- and (S_p)-**11** in hexane (1.0 × 10^{-5}) excited at 300 nm (for CPL) and at each absorption maximum (for PL).

(A) CD and UV-vis absorption spectra in cyclohexane (B) CPL and PL spectra in cyclohexane

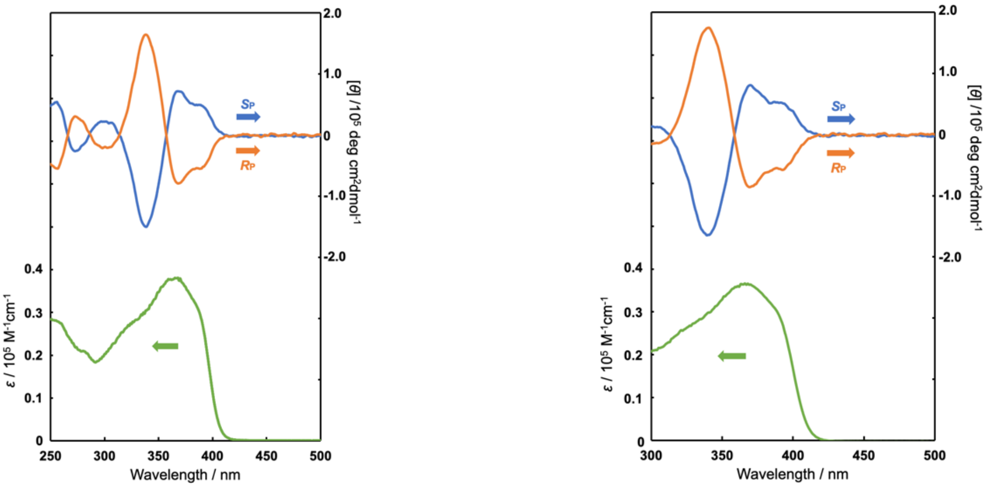

$\lambda_{PL,max}$ = 541 nm, $|g_{lum}|$ =1.6 × 10^{-4}

Figure 3. (A) CD and UV-vis absorption spectra of (R_p)- and (S_p)-**11** in cyclohexane (1.0 × 10^{-5}). (B) CPL and PL spectra of (R_p)- and (S_p)-**11** in cyclohexane (1.0 × 10^{-5}) excited at 300 nm (for CPL) and at each absorption maximum (for PL).

(A) CD and UV-vis absorption spectra in toluene (B) CPL and PL spectra in toluene

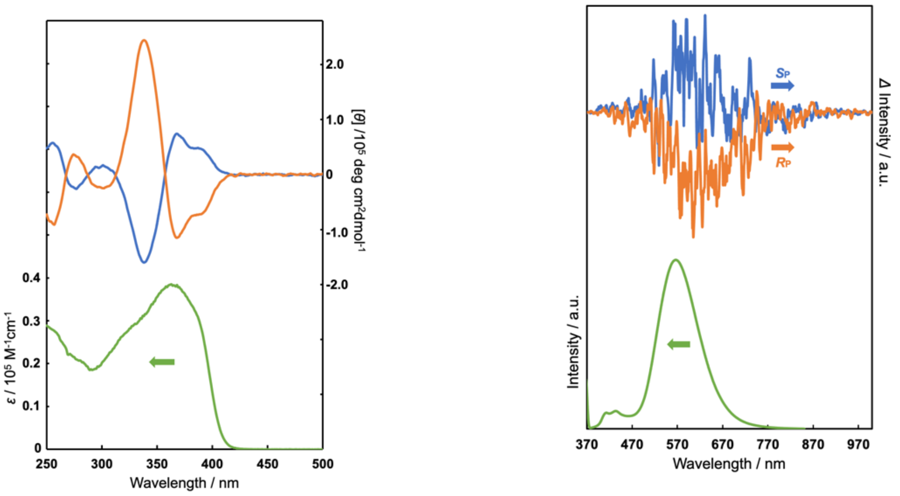

$\lambda_{PL,max}$ = 591 nm, $|g_{lum}|$ =1.0 × 10^{-4}

Figure 4. (A) CD and UV-vis absorption spectra of (R_p)- and (S_p)-**11** in toluene (1.0 × 10^{-5}). (B) CPL and PL spectra of (R_p)- and (S_p)-**11** in toluene (1.0 × 10^{-5}) excited at 300 nm (for CPL) and at each absorption maximum (for PL).

(A) CD and UV-vis absorption spectra in 1,4-dioxane (B) CPL and PL spectra in 1,4-dioxane

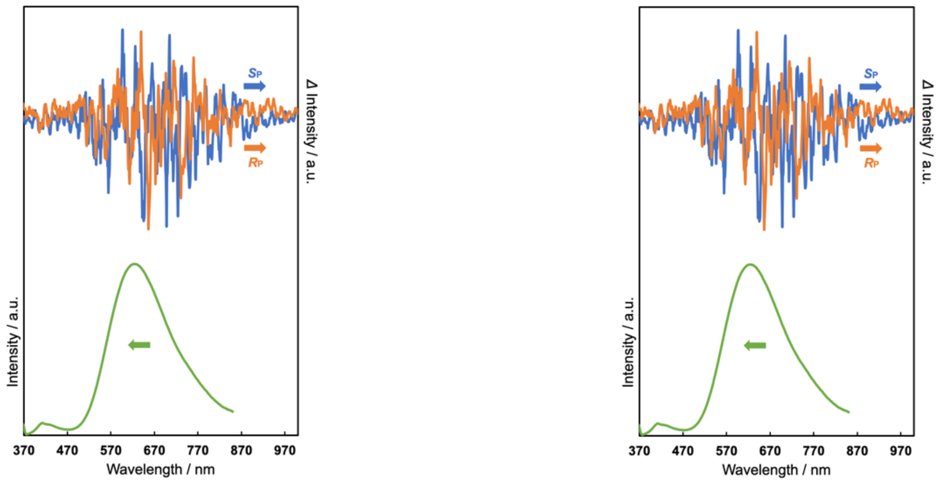

$\lambda_{PL,max}$ = 623 nm, $|g_{lum}|$ ⋯ not calculated due to the noisy spectra

Figure 5. (A) CD and UV-vis absorption spectra of (R_p)- and (S_p)-**11** in 1,4-dioxane (1.0 × 10^{-5}). (B) CPL and PL spectra of (R_p)- and (S_p)-**11** in 1,4-dioxane (1.0 × 10^{-5}) excited at 300 nm (for CPL) and at each absorption maximum (for PL).
